# Direct Imaging of Local pH Reveals Bubble-Induced Mixing in a CO_2_ Electrolyzer

**DOI:** 10.1021/acssuschemeng.3c01773

**Published:** 2023-07-03

**Authors:** Lorenz M. Baumgartner, Aron Kahn, Maxime Hoogland, Jorrit Bleeker, Wolter F. Jager, David A. Vermaas

**Affiliations:** Department of Chemical Engineering, Delft University of Technology, Van der Maasweg 9, 2629 HZ Delft, The Netherlands

**Keywords:** CO_2_ reduction, operando fluorescence imaging, gas diffusion electrode, bipolar membrane, pH imaging

## Abstract

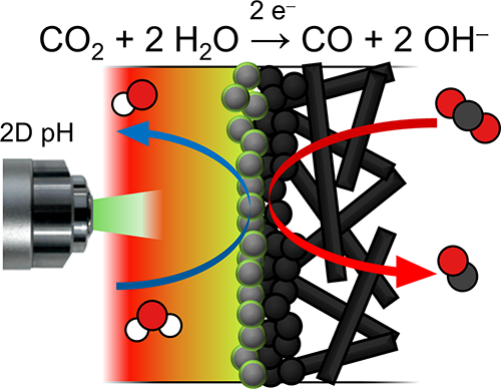

Electrochemical CO_2_ reduction poses a promising pathway to produce hydrocarbon chemicals and fuels without relying on fossil fuels. Gas diffusion electrodes allow high selectivity for desired carbon products at high current density by ensuring a sufficient CO_2_ mass transfer rate to the catalyst layer. In addition to CO_2_ mass transfer, the product selectivity also strongly depends on the local pH at the catalyst surface. In this work, we directly visualize for the first time the two-dimensional (2D) pH profile in the catholyte channel of a gas-fed CO_2_ electrolyzer equipped with a bipolar membrane. The pH profile is imaged with operando fluorescence lifetime imaging microscopy (FLIM) using a pH-sensitive quinolinium-based dye. We demonstrate that bubble-induced mixing plays an important role in the Faradaic efficiency. Our concentration measurements show that the pH at the catalyst remains lower at −100 mA cm^–2^ than at −10 mA cm^–2^, implying that bubble-induced advection outweighs the additional OH^–^ flux at these current densities. We also prove that the pH buffering effect of CO_2_ from the gas feed and dissolved CO_2_ in the catholyte prevents the gas diffusion electrode from becoming strongly alkaline. Our findings suggest that gas-fed CO_2_ electrolyzers with a bipolar membrane and a flowing catholyte are promising designs for scale-up and high-current-density operation because they are able to avoid extreme pH values in the catalyst layer.

## Abstract 2

The electrochemical CO_2_ reduction reaction depends strongly on the local pH at the cathode gas diffusion electrode. We use a 2D imaging technique to show that gas bubble evolution plays a critical role in the local pH profile and CO_2_ mass transfer.

## Introduction

Electrochemical CO_2_ reduction (CO_2_R) could be a promising process to make renewable energies more effective in mitigating climate change^1,2^ and to ensure energy security. CO_2_R could utilize electricity from renewable power sources for the sustainable production of hydrocarbon chemicals and fuels.^3^ To this end, CO_2_ can be captured from point sources,^4^ directly from the air,^5^ or the ocean,^6^ and then reduced electrochemically. Depending on the cathode catalyst, useful chemical intermediates can be formed (e.g., Ag: CO,^7^ Sn: HCOOH,^8^ or Cu: C_2_H_4_, CH_4_, or ethanol^9^).^10^ These could then be further processed into organic chemicals or hydrocarbon fuels using established industrial processes (Fischer–Tropsch or methanol synthesis).^3^

To make electrochemical CO_2_ reduction (CO_2_R) economically viable, the process has to be operated at a high current density (e.g., *j* ≥ −200 mA cm^–2^),^11^ a high Faradaic efficiency (e.g., *FE*_CO_ ≥ 95%),^12^ and a low cell potential (e.g., *E*_cell_ ≤ 3 V).^13^ The CO_2_ mass transfer to the catalyst strongly affects the *FE* for the desired carbon products (e.g., CO). If the mass transfer of CO_2_ cannot keep up with the supply of electrons at sufficiently high *j*, the excess current is consumed in the hydrogen evolution reaction (HER), leading to a decrease in *FE*_CO_. The introduction of gas diffusion electrodes (GDEs) has made it possible to maintain a high *FE* for carbon products at a high *j* by ensuring a sufficient CO_2_ mass transfer rate to the catalyst layer (CL).^14^

CO_2_ electrolysis with flowing catholyte is typically carried out with pH-neutral electrolytes such as KHCO_3_^15,16^ or, less commonly, K_2_SO_4_.^17,18^ While, for example, the bulk of a 1 M KHCO_3_ electrolyte has a CO_2_ solubility limit of 0.034 mM and a pH of 7.8, the local concentration of CO_2_ and pH at the actual catalyst surface can deviate depending on the process conditions.^19^ The local pH at the catalyst surface still affects the *FE* by changing the relative reaction rates of CO_2_R and HER. While neutral pH values are not detrimental to CO_2_R,^15,20^ highly alkaline pH values reduce the reaction rate because of the carbonate equilibrium. The local pH increases due to hydroxide formation in the CO_2_R reaction (CO_2_ + H_2_O + 2e^–^ → CO + 2OH^–^). At a sufficiently high pH, CO_2_ forms bicarbonate (CO_2_ + OH^–^ ↔ HCO_3_^–^; p*K*_a,1_ = 6.4) and carbonate (HCO_3_^–^ + OH^–^ ↔ CO_3_^2–^; p*K*_a,2_ = 10.3) in homogeneous buffer reactions.^21^ Therefore, a high local pH in the CL can diminish the *FE* for CO_2_R.^22^

Also a too low pH can have a negative impact on *FE*_CO_. Because the exchange current density of proton reduction (2H^+^ + 2e^–^ → H_2_) is 3 orders of magnitude higher compared to water reduction (H_2_O + 2e^–^ → 2OH^–^),^23^ the HER is significantly faster in acidic conditions. Therefore, *FE*_H_2__ can increase at low pH values, especially if the CO_2_R is limited by CO_2_ mass transfer.^20^ At high current densities, however, the locally higher pH near the catalyst surface can alleviate the low selectivity for CO_2_R.

In conclusion, we expect high *FE* for the desired CO_2_R products as long as there is sufficient CO_2_ mass transfer to the catalyst surface and the local pH is not too acidic or too alkaline. It can be challenging, however, to achieve these ideal conditions in practice because they are affected by many interdependent phenomena (e.g., electrochemical reactions, homogeneous reactions, and mass transfer in gas and liquid phases). For this reason, researchers have tried to gain a deeper understanding of the reaction system with numerical simulations in one dimension (1D)^24,25^ or two dimensions (2D).^22,26,27^ The reaction system is further complicated by the evolution of gas bubbles on the electrodes, which can affect the energy efficiency by introducing overpotentials.^28,29^

Experimental characterization techniques^30,31^ can be used to complement these numerical studies. For example, absorption spectroscopy can determine the pH at plate cathode surfaces^32,33^ or inside a bipolar membrane (BPM).^34^*Operando* NMR has been used to study aqueous CO_2_ electrolysis on silver or copper plate electrodes.^35,36^*Operando* Raman microscopy has allowed us to measure the local (bi)carbonate concentrations and pH values depending on the distance from the cathode in a liquid-fed^37^ or gas-fed^38^ CO_2_ electrolysis flow cell. This technique is limited by the relatively low intensity of the Raman effect,^39^ which restricts the imaging speed (typical acquisition time: ≥10 min).^40,41^ Fluorescence microscopy, in contrast, can use the strong fluorescence signal of suitable probe molecules to measure spatially resolved intensity more rapidly,^42^ allowing much shorter acquisition times (e.g., 5 s).^43^ In another example, Leenheer et al. assessed the activity of water-splitting electrocatalysts by recording 2D images with a pH-sensitive ratiometric dye.^44^

Fluorescence lifetime imaging microscopy (FLIM) is an imaging technique that uses special fluorescence lifetime probe molecules. Because these dye molecules change their fluorescence lifetime depending on their local environment (e.g., pH or concentration of certain species), FLIM can measure the corresponding spatially resolved local environment of a sample based on the fluorescence lifetime instead of the absolute intensity.^42,45^ This makes FLIM especially useful for applications in which inhomogeneous excitation or differences in dye concentration can affect the intensity. While FLIM has been predominantly used to study biological samples,^46,47^ it has also enabled the study of ion transport in electrochemical systems, in which the electromigration of charged dye molecules can lead to concentration gradients that would complicate the use of intensity-based imaging. For example, Benneker et al. used FLIM to study the mass transfer of NaCl in a microfluidic desalination cell.^48^ In another example, de Valença et al. investigated the mass transfer of Cu^2+^ ions in an electrochemical cell.^43,49^ So far, only a limited number of studies have applied FLIM to CO_2_ electrolysis. For example, Kalde et al. used FLIM to qualitatively determine electrochemically active areas in a microfluidic model of a GDE.^50^

In this work, we study the electrochemical performance and the local pH profile of a CO_2_ electrolyzer with a flowing K_2_SO_4_ catholyte and a bipolar membrane. The effects of process parameters, i.e., current density, CO_2_ saturation, and catholyte flow rate, on the Faradaic efficiency for CO are investigated. For the first time, the 2D pH profile in a CO_2_ electrolyzer catholyte channel was visualized experimentally using *operando* FLIM with a pH-sensitive quinolinium dye. This dye was recently developed by us and allows pH measurements between pH 6–9 and 11–13.^51^ We demonstrate that bubble-induced mixing plays an important role in the pH profile in the catholyte and the Faradaic efficiency.

## Experimental Methods

**Figure 1:**
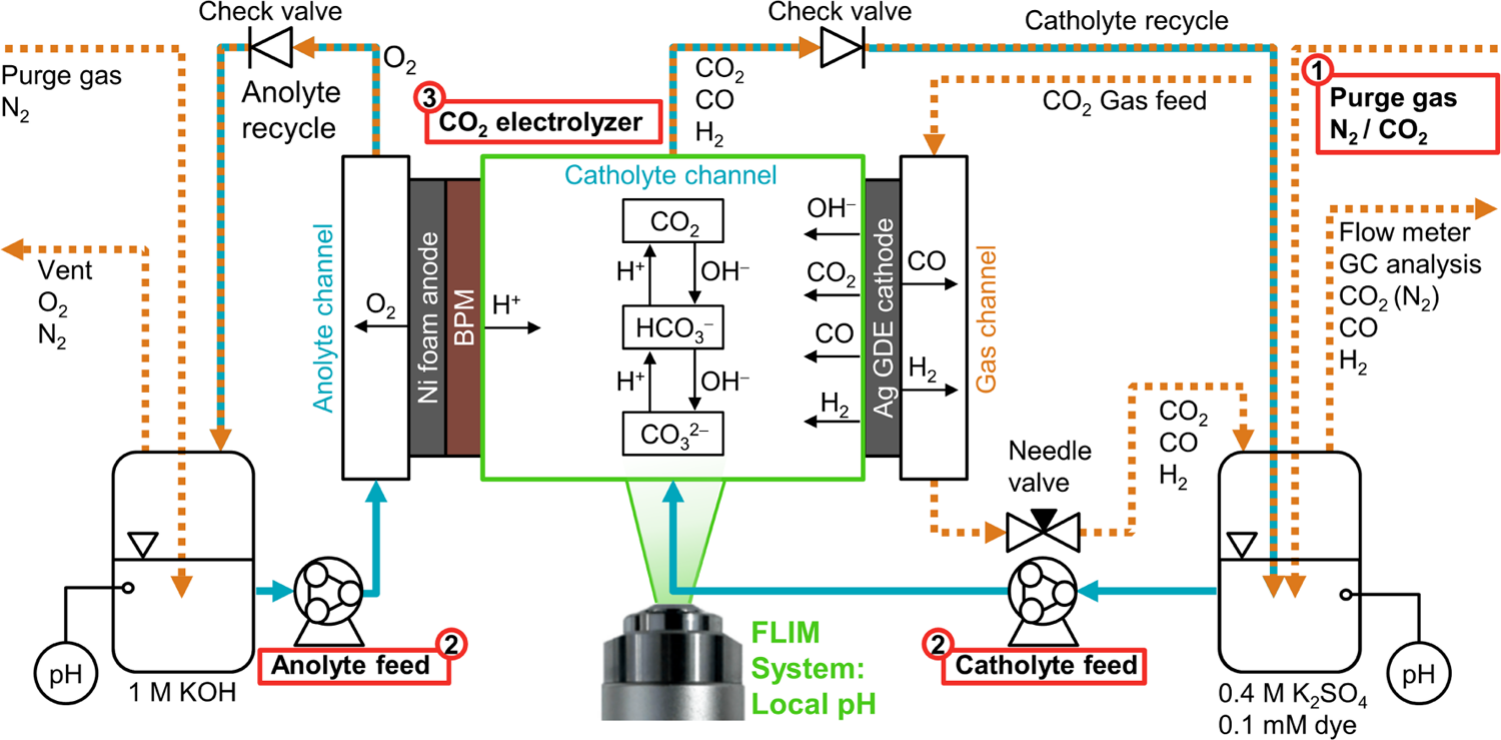
Process flow diagram of the CO_2_ electrolysis setup with *operando* FLIM of the local catholyte pH. Process parameters: (1) Catholyte purge gas: N_2_ purge or saturation with CO_2_; (2) liquid flow rate: 0.9 or 9.0 mL min^–1^ (≙ *Re* = 5 or *Re* = 50 in catholyte channel); (3) current density: −10, −50, −100 mA cm^–2^. The anolyte and the catholyte channel were separated with a bipolar membrane (BPM). The backpressure of both electrolyte streams was set by check valves. Both electrolytes were recirculated to their respective reservoirs, in which the gaseous products were removed with a purge gas. The bulk pH inside the reservoir was measured with a pH meter. The CO_2_ gas feed was humidified to 85% relative humidity (r.h.) at 20 °C and passed into the gas channel of the electrolysis cell at a flow rate of 10 mL min^–1^. The gas backpressure was controlled with a manual needle valve. The composition of the cathode product gas was analyzed with gas chromatography (GC). The flow rate was measured with a bubble flow meter. A more detailed process flow diagram is available in the Supporting Information (SI) (Figure S4).

The CO_2_ electrolysis with *operando* fluorescence lifetime imaging microscopy (FLIM) was carried out with the setup shown in Figure 1. The 3-compartment electrolysis cell (Figures S1–S3) was equipped with a porous nickel foam as the anode. The cathode GDE was prepared by depositing an Ag catalyst layer (1.0 mg Ag cm^–2^, 20 wt % Nafion) on an SGL 39BC gas diffusion layer.^52^ Its active area had a height of 25 mm and an electrode width of 4 mm (1 cm^2^). The adjacent catholyte channel had a matching depth of 4 mm. The gap width between the GDE and the BPM was 2 mm (Figure S2). The BPM separated the anolyte (1 M KOH) and catholyte (0.4 M K_2_SO_4_, 0.1 mM fluorescent dye) ensuring that no significant bulk pH change occurred. Both electrolytes were recirculated during the experiment (Figure 1). The humidified CO_2_ feed was supplied to the GDE in flow-by mode. The backpressure of the gas compartment was controlled with a needle valve. A purge gas stream was used to flush the product gases from the catholyte reservoir.

The effects of three process parameters were investigated (Figure 1): The catholyte was continuously purged (1) with N_2_ or CO_2_ purge gas. The Reynolds number (2) in the catholyte channel, *Re*, was set to 5 or 50 by adjusting the liquid flow rate (see Section S2.1 in the SI). After adapting the gas backpressure to achieve a flow-by regime at the GDE, we set a series of current densities (3) in galvanostatic mode (−10, −50, and −100 mA cm^–2^). The equilibration time for each process parameter set was 20 min. Then, we performed three measurements of the gas flow rate and three injections with a gas chromatography (GC) system to determine the Faradaic efficiency of the cathode side.

In parallel, we used the FLIM system to record a series of local pH images at three different heights of the flow cell (Figure S5). A more detailed experimental procedure is available in Section 2.2 in the SI. The FLIM system (Figure S6) used a diode laser (405 nm, 20 MHz, 300 mW) as the excitation light source. The modulated laser light passed through a spinning disk confocal unit, which uses disks with microlenses and pinholes to restrict the excitation and emission light paths to a single focal plane.^53^ The basis of the system was an inverted microscope with a 5× objective to record images with a width of 2.4 mm and a height of 2.2 mm. The microscope was focused on the center of the catholyte channel, which corresponds to a depth of 2 mm (Figure S5). The focused laser light excited the fluorescent quinolinium dye in the catholyte (0.1 mM).^51,54^ The fluorescent light emitted by the dye was filtered by the spinning disk unit and recorded with the FLIM camera (512 × 470 pixels). The camera used the frequency-domain technique to record fluorescent lifetime images.^45^ The lifetime images are calculated from 6 phase shift images, which each have an exposure time of 75 ms. This results in a total imaging time of 450 ms per frame. We calibrated the FLIM system with an in-line titration setup (Figure S6). The resulting calibration curve was used to convert fluorescent lifetime images to local pH images (Figure S7).

## Results and Discussion

We carried out a series of galvanostatic CO_2_ electrolysis experiments with *operando* FLIM to image the local pH in the catholyte channel. Supplementary calculations, additional results, and the numerical values of plotted data are included in the Supporting Information (SI).

### Fluorescence Lifetime Imaging Microscopy (FLIM) Applied to Operando CO_2_ Electrolysis

**Figure 2:**
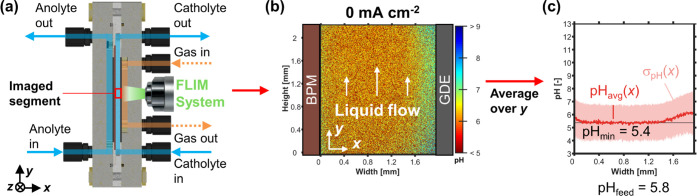
Operando FLIM validation. (a) Schematic of the flow cell: Imaging of catholyte flow channel segment in *x*–*y* plane. (b) 2D pH profile over channel height (*y*) and width (*x*). Left: The BPM produces H^+^ ions during operation. Right: The GDE is fed CO_2_ from the gas channel (not visible). (c) The 1D pH profile, pH_avg_(*x*), was averaged over the height of the channel segment. The shaded red area indicates the standard deviation of the pH value, σ_pH_(*x*). The minimum value of pH_avg_(*x*) is pH_min_. The maximum value of pH_avg_(*x*) is pH_max_. The pH value of the catholyte feed, pH_feed_, was measured with a pH meter.

For validation, the local pH of a catholyte channel segment with the dimensions of 2 × 2 mm was imaged (Figure 2a), which means one pixel covers about 5 μm × 5 μm. For a current density of 0 mA cm^–2^, the 2D pH profile in Figure 2b is obtained. The corresponding 1D pH profile is generated by averaging over the *y*-coordinate of the segment (Figure 2c). The catholyte bulk pH, pH_min_, of 5.4 ± 1.4 is in good agreement with the feed pH, pH_feed_, of 5.8, which was obtained from an independent measurement with a pH meter (Figure 2c). The standard deviation of the average pH, σ_pH_(*x*), may seem relatively large, but the 2D image (Figure 2b) shows that the noise is randomly distributed in the y-direction, which makes the profile of pH_avg_ statistically reliable. The FLIM images show an increased average pH, pH_avg_(*x*), close to the walls on both sides of the channel (Figure 2a). This is a systematic error, which most likely originates from the fluorescence of the adjacent poly(ethylene terephthalate) (PET) gaskets (Figure S9), which emit a constant fluorescence lifetime corresponding to about pH 6. Because this signal is convoluted with the fluorescence response of the pH-sensitive dye in the catholyte, our images overestimate the pH at the wall when the actual pH < 6, and probably underestimate the true pH_max_ when an alkaline boundary layer forms during operation.

The FLIM results in Figure 2 demonstrate that we can map the pH in a 2 mm wide CO_2_ electrolyzer flow cell in 2D, accepting noise at the micrometer scale and an offset near the edges. The quinolinium-based dye is most sensitive to pH changes between pH 6 and 9 (Figure S7). This allows us to study the local pH near the GDE when a current is applied to the cell.

### Performance Indicators for BPM-Based CO_2_ Electrolyzers

Our electrolyzer operates with a similar performance as BPM electrolyzers with flowing catholyte reported in the literature (Table 1). The *E*_cell_ of our system is higher than that reported by Chen et al. (4.5 vs. 3.5 V) because of the wider catholyte gap, *d*_cath_ (2 vs. 1.3 mm), and lower operating temperature, *T* (20 vs. 60 °C).^18^ De Mot et al. achieved a significantly higher *FE*_CO_ of 94%.^55^ This improvement cannot be explained by their higher catalyst loading because the effect of loading on *FE*_CO_ levels off after about 1.25 mg Ag cm^–2^.^56^ Instead, the higher *FE*_CO_ can probably be attributed to the difference in catholyte. We used 0.4 M K_2_SO_4_, a neutral electrolyte without pH buffering capacity. De Mot et al., in contrast, used 0.5 M KHCO_3_,^55^ which suppresses the HER from proton reduction and can buffer the pH in the CL.^57^

**Table 1:** Electrochemical Performance of Gas-Fed CO_2_ Electrolyzers with BPM and Flowing Catholyte^a^

parameter	this work	Chen et al.^18^	De Mot et al.^55^
GDE catalyst	1.0 mg Ag cm^–2^	0.5 mg SnO_2_ cm^–2^	2.5 mg Ag cm^–2^
*j*	–100 mA cm^–2^	–100 mA cm^–2^	–100 mA cm^–2^
*FE*	CO: 70%	HCOOH: 73%	CO: 94%
catholyte	0.4 M K_2_SO_4_	0.4 M K_2_SO_4_	0.5 M KHCO_3_
pH_feed_	5.5	na	7.6
*d*_cath_	2.0 mm	1.3 mm	1.0 mm
*T*	20 °C	60 °C	60 °C
*E*_cell_	4.5 V	3.5 V	4.6 V

a The nickel anode was pressed against the BPM in zero-gap configuration and supplied with KOH anolyte. The current density is *j*. The Faradaic efficiency is *FE*. The bulk pH of the catholyte feed is pH_feed_. The thickness of the catholyte gap between BPM and cathode GDE is *d*_cath_. The electrolyzer temperature is *T*. The cell potential is *E*_cell_. This work: The *FE*_CO_ of 70% was measured for a Reynolds number, *Re*, of 5, which corresponds to a catholyte flow rate of 0.9 mL min^–1^. The catholyte was purged with N_2_ to remove dissolved CO_2_.

**Figure 3:**
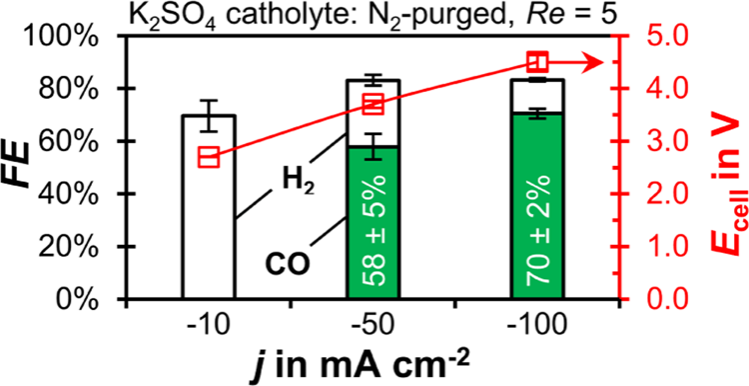
CO_2_ reduction performance for gas-fed CO_2_ electrolyzers with N_2_-purged catholyte at *Re* = 5. The *FE* for CO and H_2_ is plotted as a function of current density, *j*, on the left *y*-axis. The error bars represent the estimated standard errors of three GC injections. The cell potential, *E*_cell_, is plotted on the right *y*-axis.

It is interesting that our electrolyzer exhibits a very poor *FE*_CO_ at −10 mA cm^–2^ (Figure 3) because CO_2_ mass transfer limitation does yet not occur at such a low *j*.^58^ Further, we have previously demonstrated that this GDE model can sustain a *FE*_CO_ of 89–74% for *j* ranging from −10 to −200 mA cm^–2^ with a 1 M KHCO_3_ catholyte.^59^ Therefore, the poor *FE*_CO_ at −10 mA cm^–2^ is probably caused by differences in the local pH in the CL when using K_2_SO_4_ catholyte.

**Figure 4:**
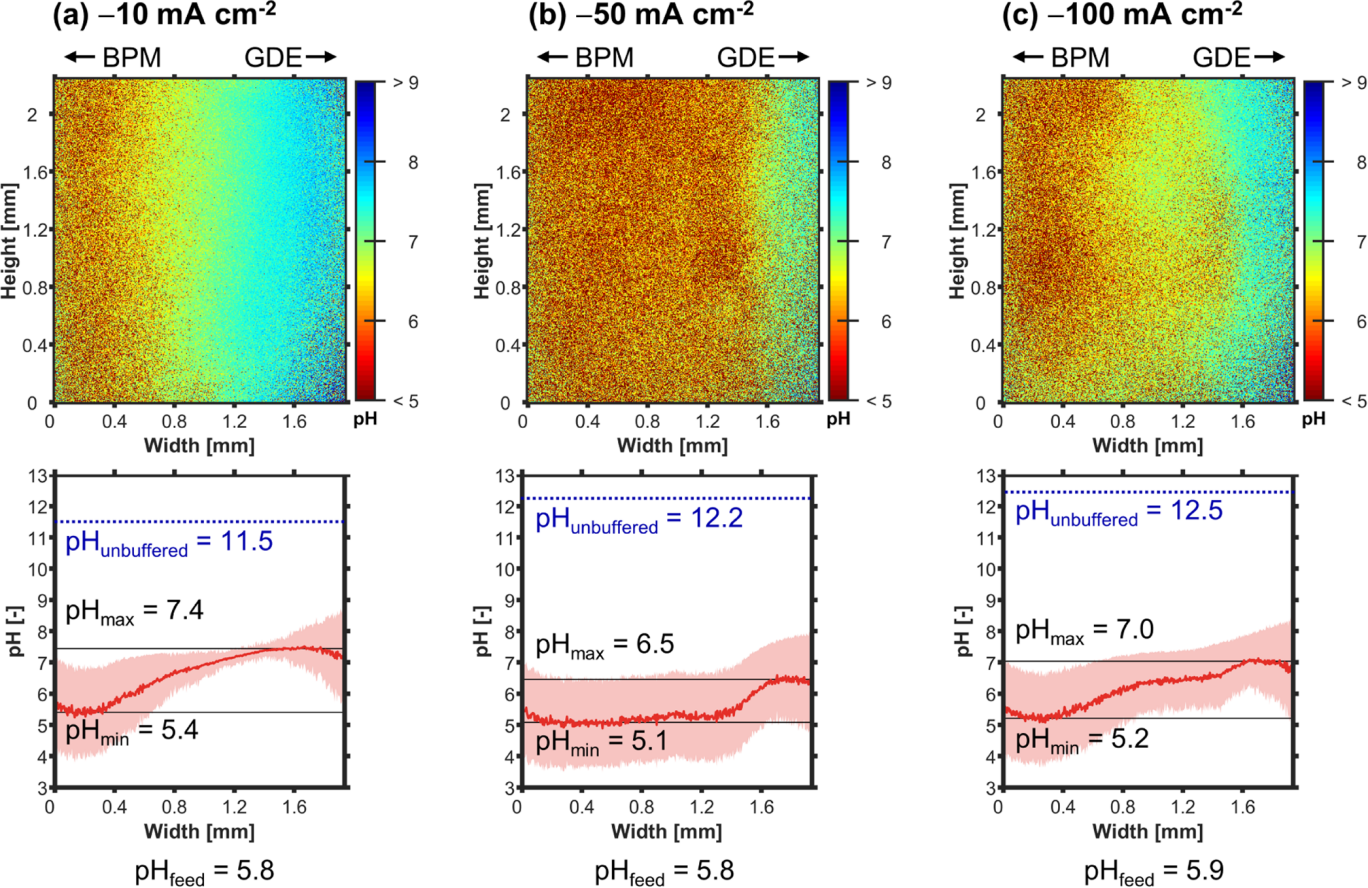
FLIM: Catholyte pH profiles at the middle of channel height (*y* = 12.5 mm) with N_2_-purged catholyte at *Re* = 5. Left: The BPM produces H^+^ ions during operation. Right: The GDE forms OH^–^ and is fed CO_2_ from the gas channel (not visible). (a–c) Effect of increasing *j*. Top: 2D pH profile over channel height and width. Bottom: The pH profile, pH_avg_, was averaged over the height of the channel segment shown in the top panel. The shaded red area indicates the standard deviation of the pH value. The minimum value of pH_avg_ is pH_min_. The maximum value of pH_avg_ is pH_max_. The pH value of the catholyte feed, pH_feed_, was measured with a pH meter. The blue dotted line indicates the value of the unbuffered pH limit, pH_unbuffered_, which we would expect if no neutralization with H^+^ occurred, no homogeneous buffering reactions took place, and OH^–^ was evenly mixed across the channel’s width (see Section 3.2 in the SI).

We expect the K_2_SO_4_ catholyte in this experiment to offer minimal pH buffering (Figure 3), especially when the catholyte is purged with N_2_, removing dissolved CO_2_ and preventing homogeneous buffering reactions in the bulk of the liquid. Therefore, the catholyte can undergo more extreme pH changes, which could lead to poor conditions for the CO_2_R in the CL. For example, CO_2_ from the gas phase might neutralize the OH^–^ produced at the catalyst surface by forming HCO_3_^–^ and CO_3_^2–^. Then, the H^+^ produced at the BPM could net acidify the catholyte.^18^ On the other hand, the OH^–^ formation inside the CL might instead lead to a locally high pH if the removal of ionic product species (OH^–^, HCO_3_^–^, and CO_3_^2–^) by the catholyte is too slow.^60^ To clarify the effect of low current density on the local pH in the CL, we analyze the catholyte pH profiles through FLIM for different current densities (Figure 4).

### Bubble-Induced Mixing Limits pH Increase and Enhances Mass Transfer

At −10 mA cm^–2^, the FLIM images show that the flowing catholyte prevents the acidification of the GDE (Figure 4a). Instead, we see the development of an alkaline boundary layer at the GDE, which originates from the OH^–^ released by the electrochemical reaction in the CL.^19^ Among all of the current densities, −10 mA cm^–2^ exhibits the highest local pH close to the CL (Figure 4a: pH_max_ = 7.4).

At −50 mA cm^–2^, the boundary layer is significantly thinner and pH_max_ is lower despite the 5x increase in OH^–^ formation rate (Figure 4b). Further, we observe the evolution of H_2_ and/or CO bubbles at the cathode surface (Figure S11). We hypothesize that the growth, break-off, and the wake flow of these bubbles lead to bubble-induced mixing between the boundary layer and the bulk of the catholyte.^61,62^ This additional mass transfer mechanism enhances the removal of product ions from the CL, which decreases pH_max_ to 6.5 (Figure 4b).

Gas evolution at electrodes influences the overpotentials of the electrolysis cell.^28,63^ We use this effect to compare the bubble formation rate for increasing *j* (Figure S12). At −10 mA cm^–2^, we observe an average of 1 bubble being released every 2 min (Figure S12a). Bubbles form at such a low frequency because the formation of dissolved products (cathode: H_2_ and CO) is relatively slow compared to the diffusion into the gas channel or into the bulk of the flowing catholyte, which leads to a low degree of oversaturation. At higher *j*, the oversaturation rises due to the more rapid formation of products, which increases the rate of nucleation, growth, and release of bubbles at the cathode.^64,65^ For example, at −50 mA cm^–2^, we estimate that around 20 bubbles are released every 2 min (Figure S12b) Therefore, bubble-induced mixing plays a much more important role at *j* = −50 mA cm^–2^ and beyond.

We hypothesize that the local pH in the porous CL has to be sufficiently close to the p*K*_a_ of the bicarbonate reaction for high *FE*_CO_. This condition is necessary to ensure that CO_2_ can be available as a dissolved gas. Due to the limitations of our technique, the pH in the CL cannot be measured directly. However, we can try to use pH_max_ as an approximation. At −10 mA cm^–2^, for example, pH_max_ reaches a value of 7.4, which might be too high compared to p*K*_a,1_ = 6.4. The true pH directly next to the GDE is probably higher than 7.4 because the fluorescence of the PET gasket makes the value appear closer to pH 6. As a result of the high local pH, this experiment yields a poor *FE*_CO_ of 0%. At −50 mA cm^–2^, the pH_max_ of 6.5 is more favorable and *FE*_CO_ rises from 0 to 58% (Figure 3). We note that for unbuffered electrolytes (e.g., K_2_SO_4_), the equilibrium pH of a CO_2_-saturated solution lies close to p*K*_a,1_ = 6.4. For buffered electrolytes, the equilibrium pH is higher (e.g., 1 M KHCO_3_: pH = 7.8), which probably influences the local availability of CO_2_ in the CL.^19^

At −100 mA cm^–2^, the thickness of the pH boundary layer and pH_max_ increase again (Figure 4c). We also observed increased bubble flow in the catholyte, which leads to the inhomogeneity in the 2D pH profile. The OH^–^ formation rate in the CL is directly proportional to *j*. In contrast, we assume that the bubble-induced mass transfer is roughly proportional to *j*^0.5^; however, it is challenging to provide an explicit relationship (see next paragraph). Based on this assumption, we suspect that the formation of OH^–^ in the CL outpaces the removal through bubble-induced mixing at sufficiently high *j*. For this reason, pH_max_ in the catholyte is higher at −100 mA cm^–2^ than for the −50 mA cm^–2^ case (Figure 4b vs. c: 6.5 vs. 7.0). It is interesting that the corresponding *FE*_CO_ increases from 58 to 70% (Figure 3). Perhaps the higher pH in the CL suppresses the HER by slowing down the proton reduction. At the same time, the local pH might not yet be so high that dissolved CO_2_ is fully converted to HCO_3_^–^ (p*K*_a,1_ = 6.4).

It is difficult to accurately predict the mass transfer coefficient for bubble-induced mass transfer. The different correlations in the literature^61,66,67^ assume that the Sherwood number for bubble-induced mass transfer, *Sh*_B_, is proportional to the square root of the Reynolds number for gas evolution, *Re*_B_ (*Sh*_B_ ∝ *Re*_B_^0.5^). *Re*_B_^0.5^ is also assumed to be proportional to *j*^0.5^; however, the different models^61,67,68^ also make assumptions about empirical parameters (e.g., bubble coverage or geometry) that are also a function of *j*. Other limitations are that the correlations were often developed for a specific set of mechanisms (e.g., nonsteady diffusion,^66,69^ or bubble release^67,68^) or match the experimental data poorly at *j* ≤ 200 mA cm^–2^.^61,62^

**Figure 5:**
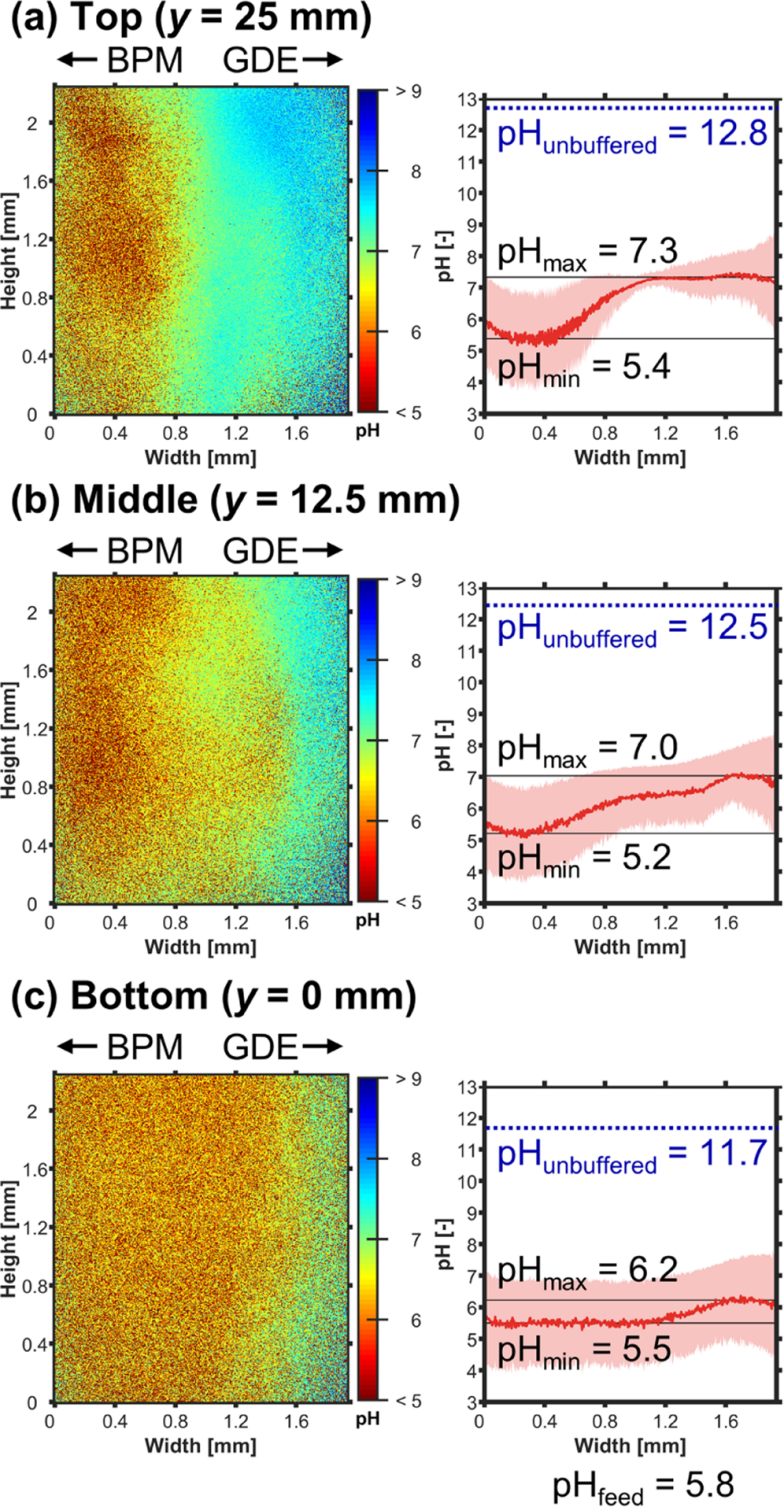
FLIM: Catholyte pH profile over the height of the electrolyzer at *j* = −100 mA cm^–2^ with N_2_-purged catholyte at *Re* = 5. (a–c) pH profiles at different *y*-positions. Left: 2D pH profile over channel height and width. Right: The pH profile, pH_avg_, was averaged over the height of the channel segment shown in the left panel. The shaded red area indicates the standard deviation of the pH value. The minimum value of pH_avg_ is pH_min_. The maximum value of pH_avg_ is pH_max_. The pH value of the catholyte feed, pH_feed_, was measured with a pH meter. The blue dotted line indicates the value of the unbuffered pH limit, pH_unbuffered_, which we would expect if no neutralization with H^+^ occurred, no homogeneous buffering reactions took place, and the OH^–^ was evenly mixed across the channel’s width (see Section 3.2 in the SI).

For all current densities, the pH_max_ in the catholyte remains far below pH_unbuffered_ (Figure 4). This is the pH limit we would expect if (1) the released OH^–^ was evenly mixed across the channel’s width, (2) no neutralization with H^+^ occurred, and (3) no homogeneous buffering reactions with CO_2_ took place (see Section 3.2 in the SI). In reality, the CO_2_ diffusing to the CL from the gas channel must result in a significant buffering of the pH by forming HCO_3_^–^ and CO_3_^2–^ species. This is clearly visible at *j* = −10 mA cm^–2^ because there is little bubble-induced mixing to facilitate the neutralization with H^+^ (Figure 4b). At *j* ≥ −50 mA cm^–2^, the H^+^ released at the BPM leads to significant acidification of the catholyte, which is visible by the drop of pH_min_ (Figure 4a vs. b: 5.4 vs. 5.1). Further, the mixing and neutralization of H^+^ and OH^–^ flattens the pH profile (Figure 4b,c). The release of H^+^ is likely to cause an even stronger (local) acidification than pH_min_ = 5.1, but this cannot be resolved with our FLIM dye, which has a plateau in the calibration curve for pH ≤ 5 (Figure S7). We can follow the development of the boundary layers at −100 mA cm^–2^ by looking at the different positions in the cell (Figure 5).

As the catholyte flows upward and accumulates OH^–^, pH_max_ increases along the height of the reactor (Figure 5a vs. c: 6.2 vs. 7.3). Because the increasing boundary thickness slows down the removal of OH^–^, we can expect the local pH in the upper parts of the CL to become less favorable for CO_2_R. To illustrate, the value of pH_max_ (*y* = 25 mm) at −100 mA cm^–2^ is similar to pH_max_ (*y* = 12.5 mm) at −10 mA cm^–2^ (Figure 5a vs. Figure 4b: 7.3 vs. 7.4), which had a *FE*_CO_ of 0%. This implies that scaled-up electrodes would have a poor local *FE*_CO_ because the top part of the electrode would mostly be producing H_2_.^22,27^ However, the observed pH_max_ is still significantly lower than the expected pH_unbuffered_ along the height of the channel (Figure 5). This raises the question to what extent the supply of CO_2_ from the GDE is able to buffer the increase of pH_max_. To deconvolute the effect of current density, forced convection, and CO_2_ saturation, we studied the cases of saturating the catholyte feed with CO_2_ and increasing the flow rate.

### CO_2_ Saturation Limits pH Increase and Enhances *FE*_CO_

**Figure 6:**
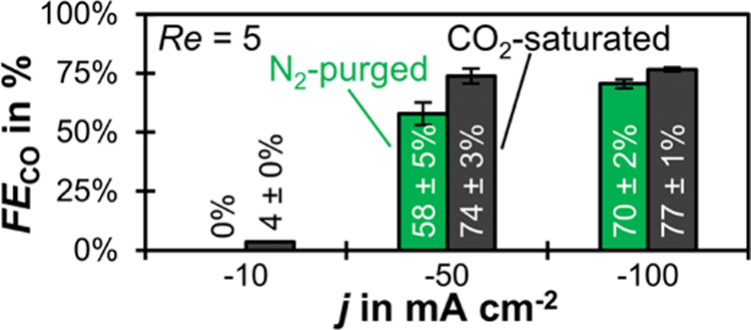
Effect of CO_2_ saturation: *FE*_CO_ as a function of *j* for CO_2_-saturated and N_2_-purged catholyte at *Re* = 5. The error bars represent the estimated standard errors of three GC injections.

Saturating the catholyte feed with CO_2_ improves the *FE*_CO_ at all investigated current densities (Figure 6). For example, we see an increase from 70 to 77% at −100 mA cm^–2^. This improvement can not be solely explained by the convective mass transfer of CO_2_ from the saturated electrolyte bulk, which constitutes an additional partial current density for CO of −2 mA cm^–2^ (see Section 3.4 in the SI). In the case of limiting CO_2_ mass transfer at −100 mA cm^–2^, this accounts for an increase in *FE*_CO_ from 70 to 72%. This suggests that there are other important mechanisms improving *FE*_CO_, such as a difference in local pH changing the relative reaction rates in the CL.

**Figure 7:**
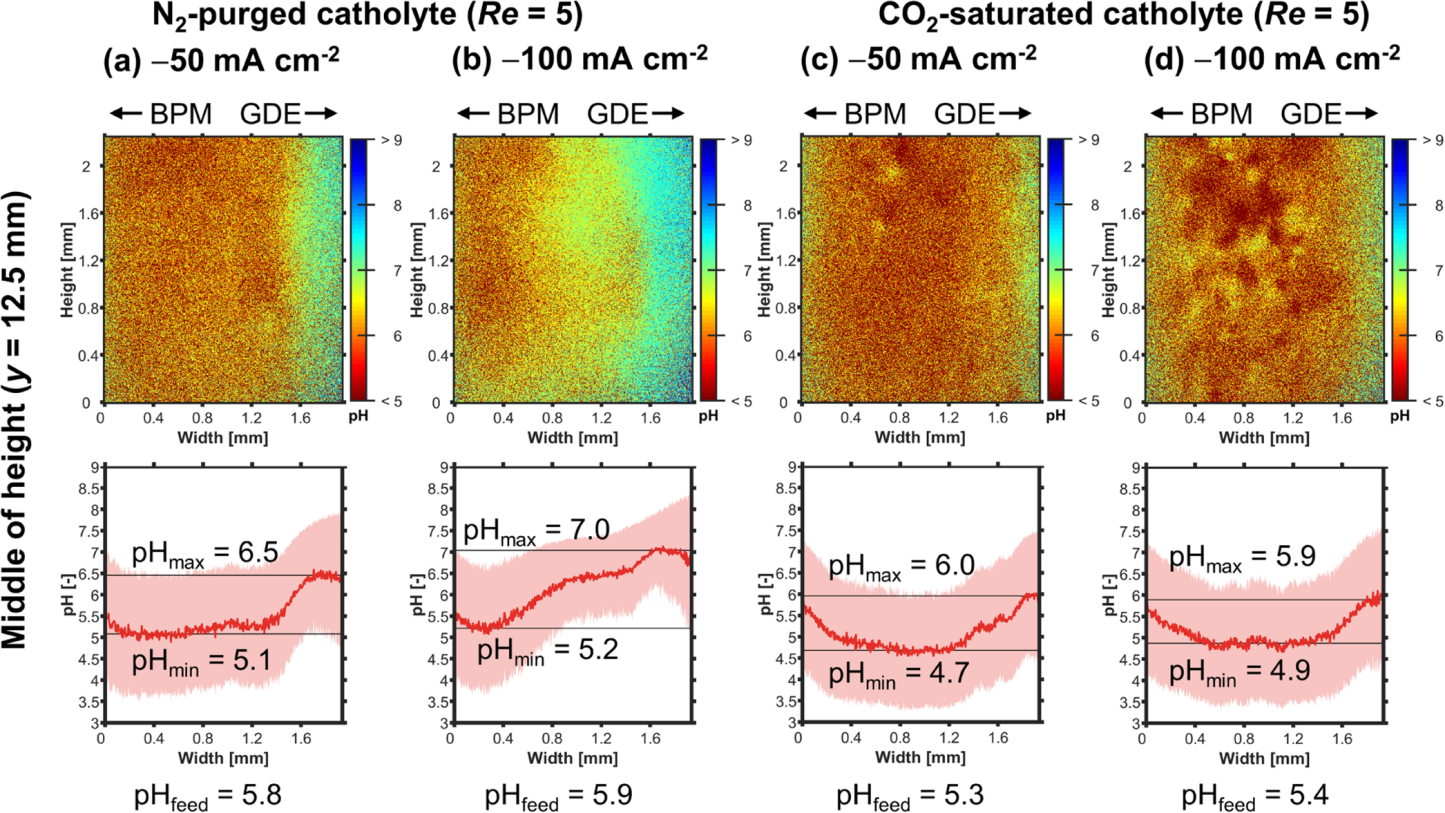
FLIM: Effect of CO_2_ saturation on catholyte pH profiles at the middle of channel height (*y* = 12.5 mm) at *Re* = 5. (a, b) Profiles for N_2_-purged catholyte with increasing *j*. (c, d) Profiles for CO_2_-saturated catholyte. Top: 2D pH profile over channel height and width. Bottom: The pH profile, pH_avg_, was averaged over the height of the channel segment shown in the top panel. The shaded red area indicates the standard deviation of the pH value. The minimum value of pH_avg_ is pH_min_. The maximum value of pH_avg_ is pH_max_. The pH value of the catholyte feed, pH_feed_, was measured with a pH meter.

The saturation with CO_2_ reduces pH_max_ at all current densities (Figure 7). For example, at −100 mA cm^–2^, pH_max_ drops from 7.0 to 5.9 (Figure 7b vs. d). This happens through multiple mechanisms. First, the dissolved CO_2_ acidifies the electrolyte by forming carbonic acid, which dissociates further into H^+^ and HCO_3_^–^. This is illustrated by the decrease in pH_feed_ from 5.8 to 5.3 (Figure 7a vs. c). Second, the dissolved CO_2_ acts as a pH buffer by forming (bi)carbonate ions. This is significant because, e.g., the CO_2_ in catholyte bulk could absorb 96% of the OH^–^ released at −100 mA cm^–2^ (see Section 3.5 in the SI). Third, the CO_2_-saturated catholyte releases CO_2_ bubbles at the BPM (Figure S14),^34,70^ which likely increases the bubble-induced mixing. Together with bubbles released at the cathode (CO, H_2_), these CO_2_ bubbles lead to the inhomogeneities seen in the 2D pH profiles (Figure 7c vs. d). Although we can not measure this effect directly, we assume that the bubble-induced mixing limits the increase of pH_max_ by enhancing the removal of product ions and also boosts the CO_2_ flux from the catholyte bulk to the CL. In summary, saturating the catholyte with CO_2_ improves *FE*_CO_ by making the local environment in the CL more favorable for CO_2_R.

### Catholyte Reynolds Number Interferes with Bubble-Induced Mixing

**Figure 8:**
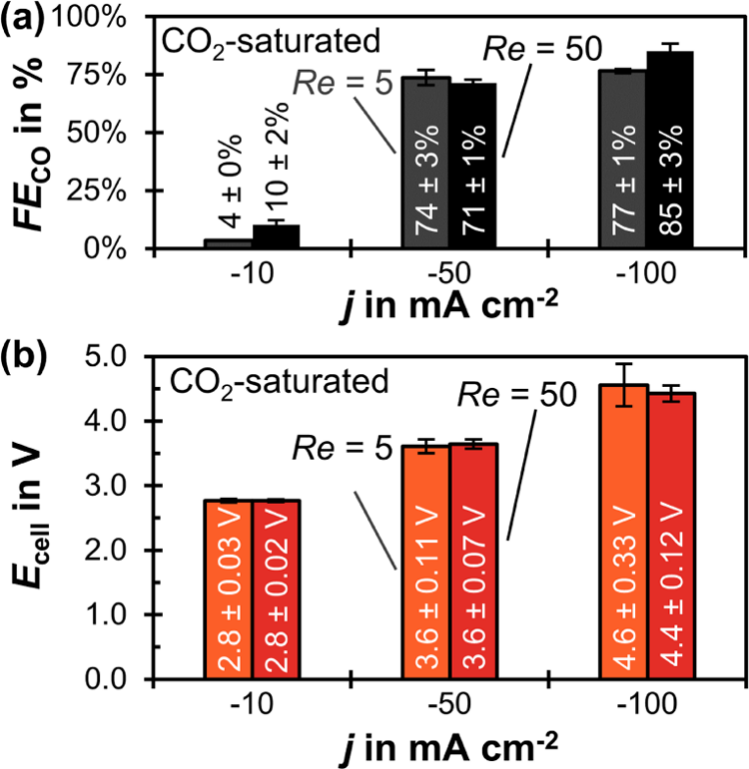
Effect of Reynolds number in catholyte channel, *Re*, for CO_2_-saturated catholyte. (a) *FE*_CO_ as a function of *j* and *Re*. The error bars represent the estimated standard errors of three GC injections. (b) Average cell potential, *E*_cell_, as a function of *j* and *Re*. The error bars represent the sample standard deviation. These potential values are calculated from the last 10 min of each electrolysis step.

The effect of additionally increasing the catholyte Reynolds number, *Re*, on *FE*_CO_ is less straightforward. At −100 mA cm^–2^, *FE*_CO_ improves from 77 to 85% when *Re* is increased from 5 to 50 (Figure 8a). This might be due to additional convective CO_2_ flux from the bulk of the catholyte. This flux is enhanced by a factor of 3.2 when *Re* is increased by a factor of 10 (*Sh* ∝ *Re*^0.5^). This increased CO_2_ flux can sustain an additional *j*_CO_ of −4 mA cm^–2^ (see Section 3.4 in the SI), which would correspond to an increase in *FE*_CO_ from 77 to 81% *FE*_CO_. There might also be important other mechanisms, such as the change in local pH, possibly explaining the observed increase to 85% *FE*_CO_. However, no significant improvement of *FE*_CO_ occurs at −50 mA cm^–2^ for the CO_2_-saturated case (Figure 8a). It is plausible that the effect of *Re* also depends on *j* because both process parameters can influence the local pH and the mass transfer.

**Figure 9:**
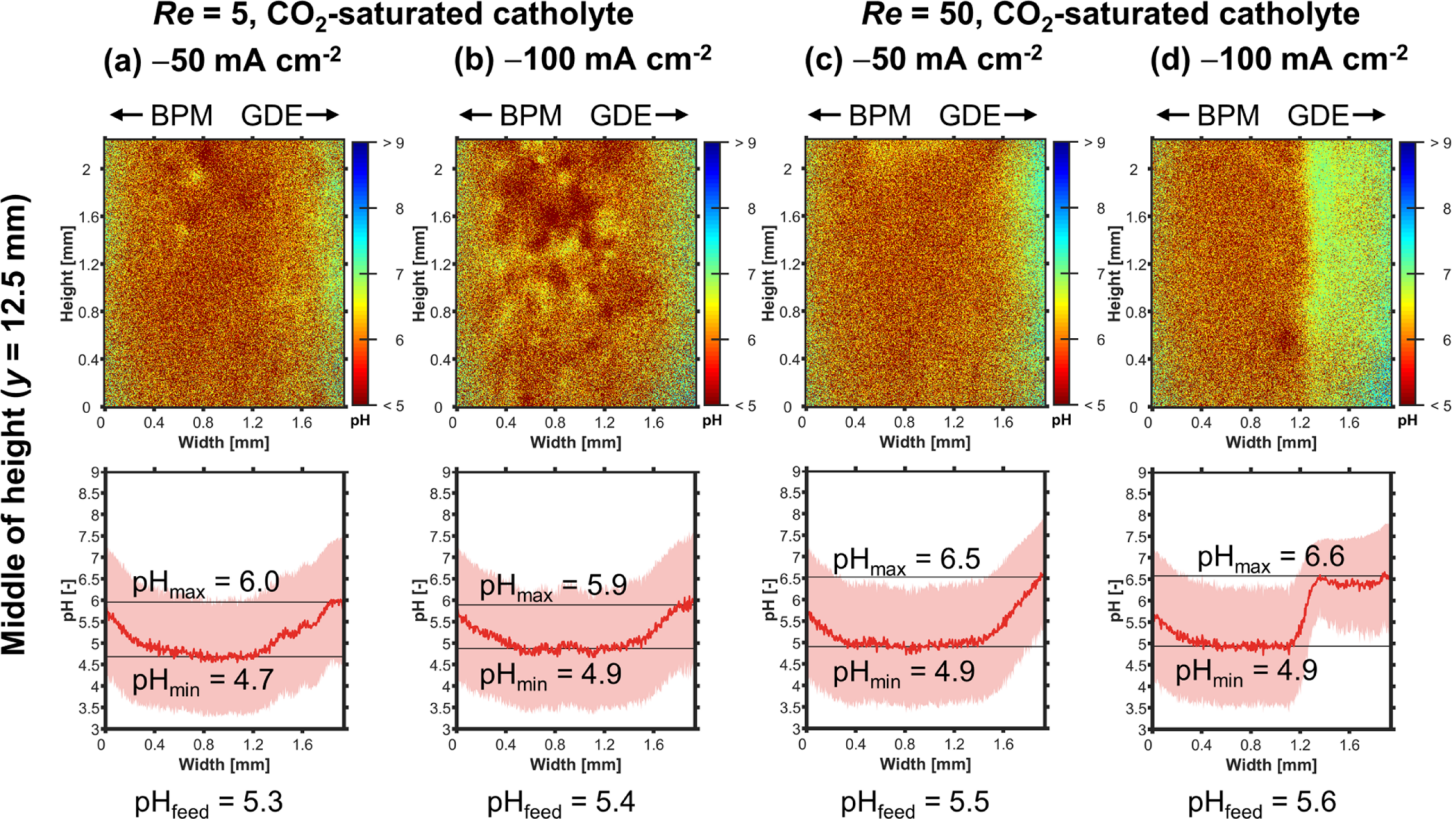
FLIM: Effect of Reynolds number, *Re*, on pH profiles at the middle of channel height (*y* = 12.5 mm) with CO_2_-saturated catholyte. (a, b) Profiles for *Re* = 5 with increasing *j*. (c, d) Profiles for *Re* = 50. Top: 2D pH profile over channel height and width. Bottom: The pH profile, pH_avg_, was averaged over the height of the channel segment shown in the top panel. The shaded red area indicates the standard deviation of the pH value. The minimum value of pH_avg_ is pH_min_. The maximum value of pH_avg_ is pH_max_. The pH value of the catholyte _feed_, pH_feed_, was measured with a pH meter.

Increasing *Re* can diminish the overpotentials caused by bubble evolution^28^ in the electrolysis cell (Figure 8b). This effect is the strongest at −100 mA cm^–2^, for which *E*_cell_ drops from 4.6 to 4.4 V when *Re* rises from 5 to 50 (Figure 8b). The reduction in overpotentials also leads to lower potential fluctuations for all *j* (Figure 8b). This effect occurs because the higher volumetric flow rate lowers the bubble nucleation rate. Bubbles evolve less frequently because dissolved product gases (e.g., CO or H_2_) are removed more quickly, which reduces their oversaturation level.^71^ In addition, the higher shear stress speeds up the release from the electrode surface.^72^ As a result, bubbles form at a lower frequency and are released with smaller diameters for a higher *Re*.^71^ Therefore, a higher liquid flow rate (*Re*) can reduce overpotentials introduced by the evolution of gas bubbles and reduce the energy efficiency of the CO_2_ electrolyzer. At the process level, however, this benefit has to be weighed against the additional pumping power required to impose the higher liquid flow rate. The optimization problem is further complicated by the effects of *Re* on *FE*_CO_ and the mass transfer.^71,72^ We can further investigate the mass transfer phenomena in the catholyte gap with snapshots of the local pH profiles (Figure 9).

To our surprise, increasing *Re* from 5 to 50 results in a higher local pH at the GDE. At −100 mA cm^–2^, for example, pH_max_ rises from 5.9 to 6.6 (Figure 9b vs. d). This is counter-intuitive because we would expect the increased forced convection to accelerate the removal of OH^–^. We hypothesize that the higher catholyte *Re* reduces the contribution of bubble-induced mass transfer. The 10× higher liquid flow rate exerts stronger drag forces on bubbles, which hinders their lateral motion. Therefore, rising bubbles are confined closer to the surface of the electrodes and less bubble mixing perpendicular to the catholyte flow direction occurs.^73^ This claim is supported by our 2D pH profiles showing less disturbances through bubbles and a more laminar flow profile when comparing *Re* = 50–5 (Figure 9d vs. b).

At −50 mA cm^–2^, increasing *Re* from 5 to 50 lowers the disturbance of the liquid flow by gas bubbles, which can be seen by the lower potential fluctuations (Figure 8b: ± 0.11 vs. ± 0.07 V). We assume this leads to a reduction in bubble-induced CO_2_ mass transfer from the catholyte, which is not sufficiently compensated by the additional mass transfer of CO_2_ through forced convection. Therefore, the CO_2_ mass transfer stagnates and *FE*_CO_ does not change significantly (Figure 8a: 74 ± 3 to 71 ± 1%). We hypothesize that the CO_2_ mass transfer is more important than the local pH for both cases because their pH_max_ is close to the p*K*_a_ of the bicarbonate reaction (Figure 9a or c: pH_max_ = 6.0 or 6.5 vs. p*K*_a,1_ = 6.4).

Increasing *j* −50 to −100 mA cm^–2^ at *Re* = 50 raises the gas evolution rate in the electrolyzer, which also results in stronger potential fluctuations (Figure 8b: ± 0.07 vs. ± 0.12 V). We assume that the higher gas evolution rate enhances the contribution of bubble-induced mixing to mass transfer thereby preventing a significant change to pH_max_ despite the higher OH^–^ formation rate (Figure 9c vs d: pH_max_: 6.5 vs. 6.6). Although pH_max_ does not change significantly, *FE*_CO_ raises from 71 to 85% (Figure 8a). This result implies that the local pH is not the only condition affecting *FE*_CO_. We hypothesize that *FE*_CO_ rises because the additional bubble mixing also enhances the CO_2_ mass transfer from the bulk. The importance of the CO_2_ mass transfer from the catholyte bulk is further highlighted by comparing the effect of *Re* between the CO_2_-saturated and the N_2_-purged cases (Figure 8a vs. Figure S15). If *j* is increased from −50 to −100 mA cm^–2^ at *Re* = 50 for the N_2_ case, no significant change of *FE*_CO_ occurs (Figure S15: 70 ± 2 to 73 ± 1%).

### Intermediate pH and High Current Density Lead to High Faradaic Efficiency

**Figure 10:**
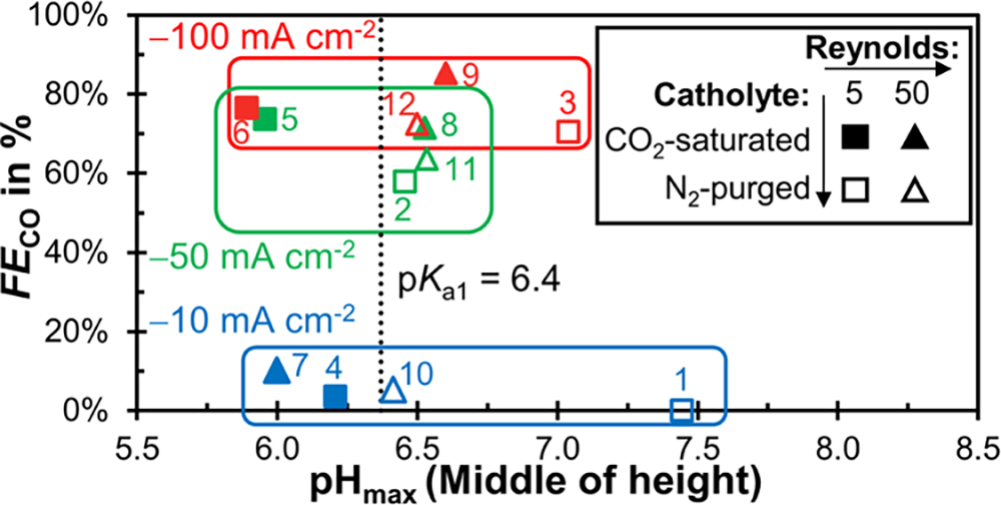
Faradaic efficiency for CO, *FE*_CO_, as a function of the maximum pH, at the middle height of the catholyte channel, pH_max_. The current density, *j*, is represented by the color of the data points (blue: −10 mA cm^–2^, green: −50 mA cm^–2^, red: −100 mA cm^–2^). The Reynolds number of the catholyte, *Re*, is indicated by the marker shape (square: *Re* = 5, triangle: *Re* = 50). Experiments with CO_2_-saturated catholyte have filled markers. The dotted line represents the p*K*_a_ of the bicarbonate reaction (CO_2_ + OH^–^ ↔ HCO_3_^–^; p*K*_a,1_ = 6.4).^21^ The number next to each marker indicates the ID number (#) of each parameter set.

The scatter plot in Figure 10 shows *FE*_CO_ as a function of pH_max_ and the process parameters. We hypothesize that the following three factors are critical to ensure a high *FE*_CO_:

- Maximum pH in the electrolyte close to the p*K*_a_ of the bicarbonate reaction (p*K*_a,1_ = 6.4): e.g., pH_max_ ≤ 7.0.
- Removal of ions from inside the porous CL to the catholyte (OH^–^, HCO_3_^–^, and CO_3_^2–^).
- Dissolved CO_2_ is available in the CL.

The CO_2_ feed in the gas channel, present in all our cases, already limits the pH_max_. Additionally, we assume that mixing induced by gas bubbles contributes to both the first (local pH in the electrolyte) and the second (local pH in CL) factor. The process parameter with the strongest effect on *FE*_CO_ is *j* because it leads to bubble-induced mixing at −50 mA cm^–2^ or higher. We think this mechanism has an important role in keeping pH_max_ close enough to p*K*_a,1_. It could further remove product ions from the CL and ensure that CO_2_ is available in the CL. For this reason, all experiments with *j* ≥ −50 mA cm^–2^ have a *FE*_CO_ ≥ 58% (Figure 10).

Saturating the catholyte with CO_2_ also has a positive effect on *FE*_CO_, which is probably due to a combination of additional pH buffering, bubble mixing, and CO_2_ mass transfer. For these reasons, the *FE*_CO_ highest values occur for experiments with CO_2_-saturated feed and *j* = −100 mA cm^–2^ (Figure 10: #9 and #6).

The effect of *Re* is less clear because the higher liquid flow rate increases the mass transfer through forced convection, but suppresses bubble-induced mixing. However, since a high *Re* seems to be beneficial for *FE*_CO_ at *j* = −100 mA cm^–2^ with a CO_2_-saturated catholyte (Figure 10: #9), this is still a relevant parameter for process optimization. It might be interesting, for example, to have a more quantitative study on how *Re* influences the dynamics of bubble growth, release, and mixing.

It is remarkable that we obtain a poor *FE*_CO_ for experiments with *j* = −10 mA cm^–2^ although their pH_max_ ranges from 6.0 to 7.4 (Figure 10: #1, #4, #7, and #10). This phenomenon might be explained through significantly higher pH values inside the porous CL, which are not accessible through FLIM. It is possible that bubble-induced mixing is necessary to exchange the catholyte inside the porous CL with the catholyte from the channel. Because this mass transfer mechanism is missing at *j* = −10 mA cm^–2^, the product ions (OH^–^, HCO_3_^–^, and CO_3_^2–^) cannot be removed sufficiently fast leading to an unfavorably high pH in the CL. This hypothesis could be validated with numerical studies in the future.

Close to the GDE of our gas-fed electrolyzer, the catholyte pH remains below 7.0 for all experiments with high *j* and high *FE*_CO_ (Figure 10: e.g., #9 and #6). In contrast, close to the plate electrode of a liquid-fed electrolyzer, the pH is estimated to be above 10 at only −15 mA cm^–2^.^19^ This raises the question how the pH close to the GDE develops for our system at *j* ≥ −200 mA cm^–2^. At these conditions, the strong bubble formation leads to a turbulent two-phase flow. This complicates recording the local pH with our FLIM system due to the limited imaging speed (450 ms per image). However, we can speculate that bubble-induced mixing and neutralization with the H^+^ from the BPM can maintain a moderate local pH at the GDE for much larger current densities. This might explain why the gas-fed BPM electrolyzer of De Mot et al. can operate at −300 mA cm^–2^ while maintaining a high *FE*_CO_ > 70%.^55^

## Conclusions

We have studied how process parameters (current density, CO_2_ saturation of the electrolyte, and catholyte flow rate) affect the Faradaic efficiency of a gas-fed CO_2_ electrolyzer with flowing K_2_SO_4_ catholyte and bipolar membrane. *Operando* fluorescence lifetime imaging microscopy (FLIM) complemented these measurements by imaging the growth of an alkaline boundary layer along the cathode GDE. Three key factors limit the pH increase at the GDE to ≤ 7.0 and enable high *FE*_CO_ of 77–85%: (1) CO_2_ from the gas phase acts as pH buffer, (2) bubble-induced mixing likely enhances the mass transfer in the catholyte channel and the ion exchange between the catalyst layer and catholyte, and (3) the CO_2_-saturated catholyte acts as pH buffer and probably leads to additional bubble-induced mixing by releasing CO_2_ at the BPM.

We hypothesize the mass transfer contribution of bubble-induced mixing to be more significant than the contribution of forced convection through the flowing catholyte. The bubble-induced mixing is only effective after exceeding a threshold in current density, which makes the maximum pH at −10 mA cm^–2^ higher than at −50 or −100 mA cm^–2^. High mass transfer rates across the channel are essential to enable a neutralization of OH^–^ from the cathode with H^+^ from the BPM. This neutralization within the channel might be able to limit the pH increase at the cathode and thereby allow a high *FE*_CO_. Therefore, gas-fed CO_2_ electrolyzers with BPM are promising systems for scale-up and operation at high current densities.

## Supporting Information Available

Methods; CO_2_ electrolysis setup; gas feed; electrolytes; process parameter 1: catholyte purge gas; process parameter 2: Reynolds number/catholyte flow rate; process parameter 3: current density (Figures S1–S23) (PDF)
All data are available in a second SI file (XLSX)
