# Supplementary material for: Direct Imaging of Local pH Reveals Bubble-Induced Mixing in a CO2 Electrolyzer
Source: ACS Sustain Chem Eng. 2023 Jul 3;11(28):10430–40. doi: 10.1021/acssuschemeng.3c01773 (PMC10354799; doi:10.1021/acssuschemeng.3c01773)
Supplement: Supplementary file 1 — sc3c01773_si_001.pdf [file sc3c01773_si_001.pdf]

SUPPORTING INFORMATION

Direct imaging of local pH reveals bubble-induced mixing in a CO_2 electrolyzer

Lorenz M. Baumgartner,[a] Aron Kahn,[a] Maxime Hoogland,[a] , Jorrit Bleeker,[a] , Wolter F. Jager, David A. Vermaas*,[a]

[a] *Department of Chemical Engineering, Delft University of Technology, Netherlands*

Email: D.A.Vermaas@tudelft.nl

Supporting Information Content:

- 23 Pages
- 3 Tables
- 23 Figures

Contents

1. General information 2

2. Methods 2

2.1 CO_2 electrolysis setup 2

2.2 Fluorescence lifetime imaging microscopy (FLIM) 6

3. Supplementary results and discussion 10

3.1 Comparison of bubble evolution for N_2-purged catholyte at Re = 5 10

3.2 Calculation of unbuffered pH limit 11

3.3 CO_2 bubble evolution at bipolar membrane 13

3.4 Catholyte Reynolds number interferes with bubble-induced mixing 13

3.5 Calculation of CO_2 buffering capacity 14

3.6 Complete set of pH profiles 15

References 22

1. General information

De-ionized water was used for all experiments. Detailed experimental results are available in the accompanying **Excel file** of the supporting information.

2. Methods

This section describes the engineering and methods in more detail.

2.1 CO_2 electrolysis setup

We used a 3-compartment flow cell made in our workshop to carry out the CO_2 electrolysis experiments (**Figure S1**). All tubing connections had an outer diameter of 1/8 inch. The cell components were pressed together with four M4 bolts tightened with a torque of 2 Nm. The anode flow plate was made of stainless steel and served as a current collector (**Figure S1**). The anolyte flow channel had a width of 3 mm and a depth of 4 mm. The flow plate was sealed against the anode spacer with a butyl rubber O-ring. The 1 mm thick anode spacer aligned the nickel foam anode (Ni-4753.01, Recemat BV, Netherlands). The anode was 1 mm thick, had an estimated average pore diameter of 0.4 mm, and a porosity of 95.2%. The anode was pressed against the BPM (Fumasep FBM, Fumatech GmbH, Germany). The membrane spacer was made of PET with a thickness of 140 μm. The active area of the BPM and GDE was controlled by PET gaskets with a rectangular hole (25 mm height x 4 mm depth = 1 cm^2).

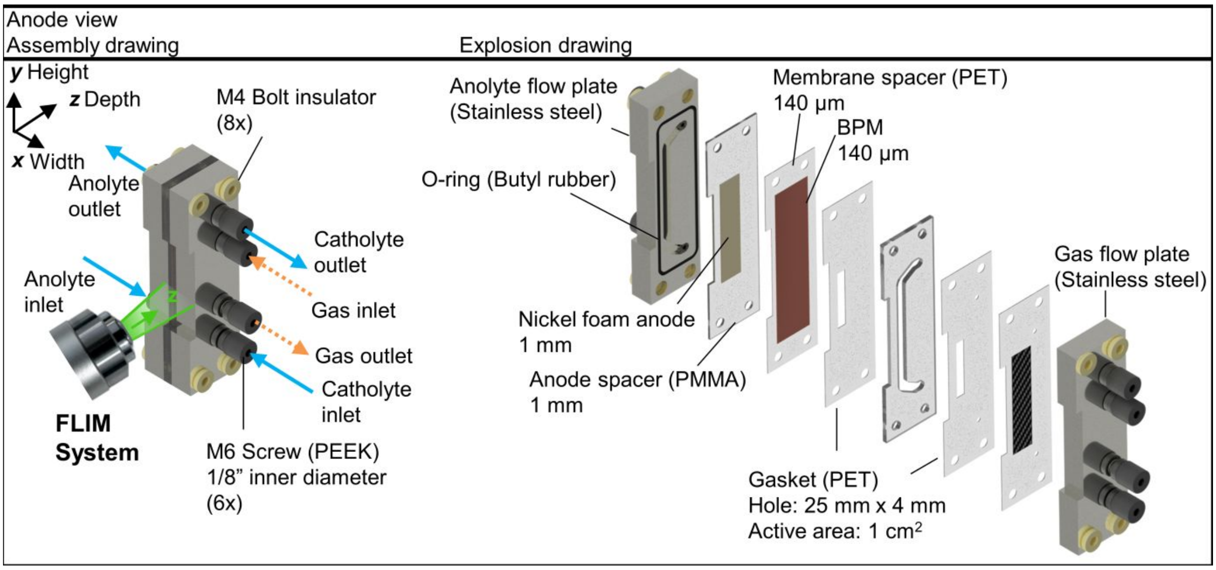

Figure S1: Anode view of the 3-compartment electrolysis flow cell.

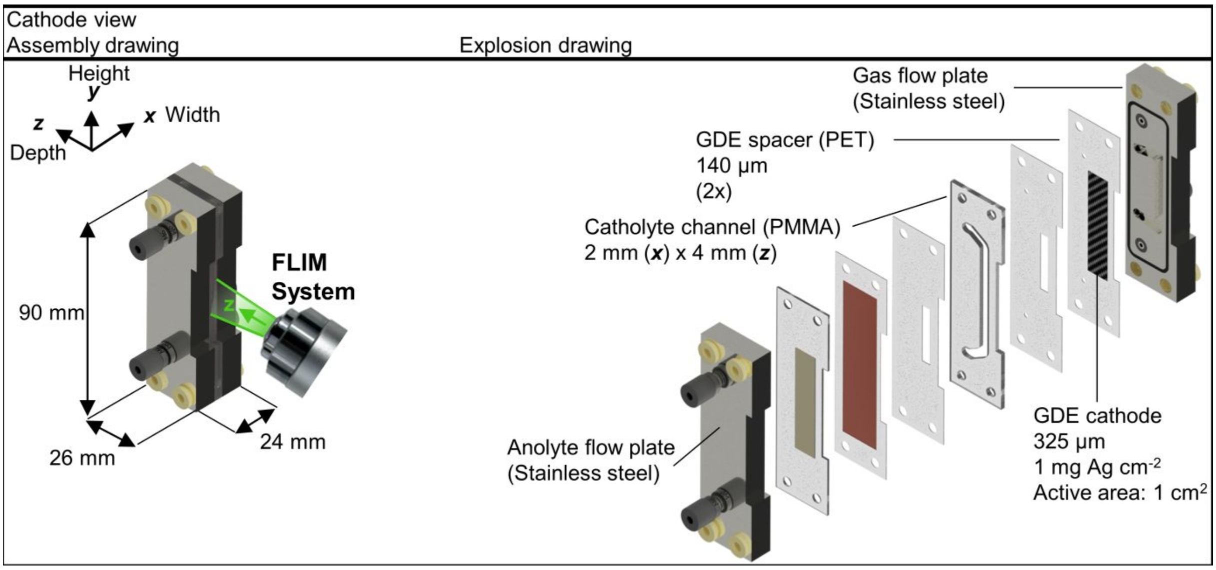

Figure S2: Cathode view of the 3-compartment electrolysis flow cell.

The catholyte flow channel had a width of 2 mm and a depth of 4 mm (**Figure S2**). The channel was cut from a PMMA sheet with a diamond milling bit to create a highly transparent surface. The GDE was manufactured by spray-coating an Ag catalyst layer on a commercial Sigracet 39BC (SGL Carbon, Germany). We described the deposition method in more detail in a previous publication.[1] The Nafion content in the catalyst layer (CL) was 20 wt%. The Ag loading was 0.94 mg Ag cm^{-2}. The GDE was aligned by two PET spacers with a thickness of 140 µm each. Assuming a GDE thickness of 325 µm, these spacers yielded a GDE compression of 14%. The gas flow plate was pressed against the GDE as a current collector. The gas flow channel had a width of 3 mm and a depth of 4 mm. The flow plate was sealed against the GDE spacer with butyl rubber O-rings.

The liquid outlets were located at the top to facilitate the removal of gas bubbles from the liquid channel (**Figure S3**). The gas outlet was located at the bottom of the cell to allow the drainage of any electrolyte that broke through the GDE into the gas channel during the start-up phase. The FLIM system recorded images of the catholyte channel in the *x*-*y* plane.

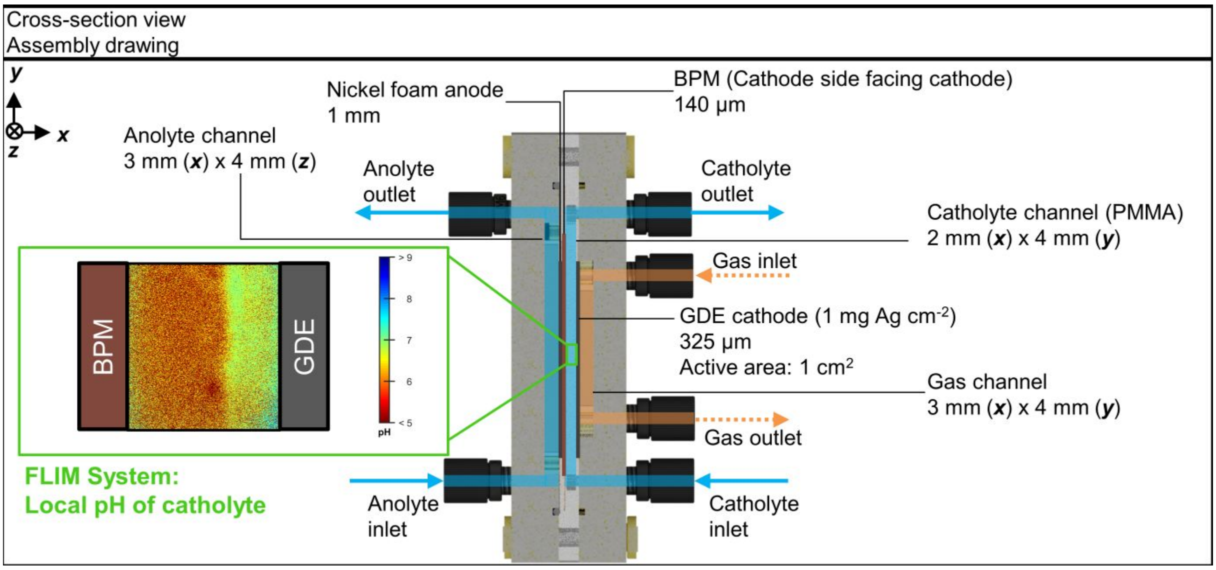

Figure S3: Cross-section view of the 3-compartment electrolysis flow cell.

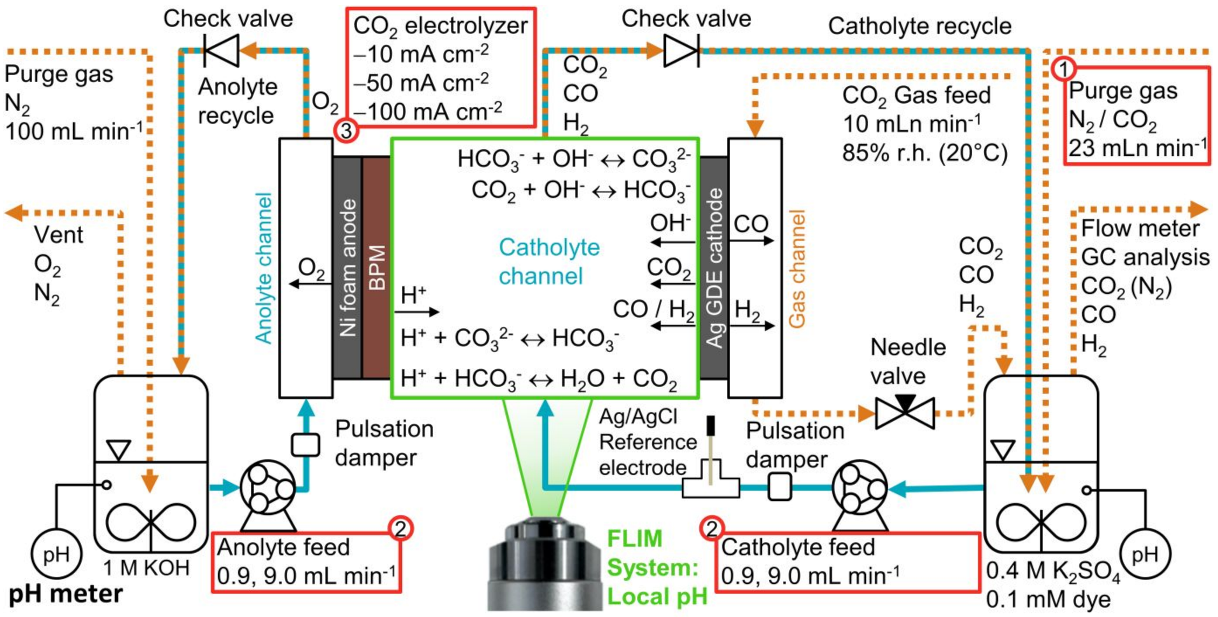

Figure S4: Process flow diagram of the CO_2 electrolysis setup with operando FLIM of the local catholyte pH. Process parameters: (1) Catholyte purge gas: N_2 purge (23 mLn min^{-1}) or saturation with CO_2 (15 mLn min^{-1}); (2) Liquid flow rate: 0.9 or 9.0 mL min^{-1} ($\triangleq$ Re = 5 or Re = 50 in catholyte channel); (3) Current density: –10, –50, –100 mA cm^{-2}. Peristaltic pumps supplied the anolyte and catholyte to the CO_2 electrolyzer. Pulsation dampers were used to reduce the pressure fluctuations caused by the pumps. The anolyte and the catholyte channel were separated with a bipolar membrane (BPM). The backpressure of both electrolyte streams was set by check valves with a cracking pressure of 69 mbar. Both electrolytes were recirculated to their respective reservoirs, in which the gaseous products were removed with a purge gas. Magnetic stirrers were used to accelerate the mixing of the electrolyte. The bulk pH inside the reservoirs was measured with a pH meter. The CO_2 gas feed was humidified to 85% relative humidity (r.h.) at 20°C inside a bubble column and passed into the gas channel of the electrolysis cell. The gas backpressure was controlled with a manual needle valve. The composition of the cathode product gas was analyzed with gas chromatography (GC). The flow rate was measured with a bubble flow meter. The cathode potential was recorded with a Ag/AgCl micro-reference electrode for the CO_2-saturated experiments.

The flow cell was installed in the CO_2 electrolysis setup (**Figure S4**). All fluid lines were made of PE. They had an outer diameter of 1/8 inch and inner diameter of 1/16 inch.

Gas feed

The CO_2 gas feed was supplied from a gas cylinder (**Figure S4**). The feed flow rate of 10 mLn min^{-1} (Normal conditions: 0°C, 1 atm) was controlled with a mass flow controller (F-201CV-020-RAD-3A-V, Bronkhorst BV, Netherlands). This flow rate corresponds to a maximum CO_2 conversion of 7% at –100 mA cm^{-2} (assuming FE_{CO} = 100%). The gas was humidified to 85% relative humidity (r.h.) at 20°C inside a bubble column.[1] and passed through the gas channel. We used a manual needle valve (SS-SS2, Swagelok, Netherlands) to control the gas backpressure. This allowed us to set a flow-by flow regime at the GDE.[2, 3]

Electrolytes

Both electrolytes were pumped with a peristaltic pump (Masterflex L/S, Cole Parmer). In-line pulsation dampers (PFD 1.06 KPZ, KNF, Switzerland) were used to dampen the flow rate fluctuations of the pump (**Figure S4**). After being pumped through the cell, the electrolytes passed through a check valve (SS-CHS2-1, Swagelok, Netherlands). The check valves had a nominal cracking pressure of 69 mbar, which set the backpressure of the electrolytes. Both electrolytes were recirculated into their storage bottles and mixed with a magnetic stirrer. The anolyte (1 M KOH) was purged with N_2 to vent the O_2 product gas and any CO_2 that might have crossed over (**Figure S4**). The purge flow rate was set to 100 mL min^{-1} with a rotameter (Reference conditions: 20°C, 1 atm).

Process parameter 1: Catholyte Purge gas

The catholyte (0.4 M K_2SO_4, 0.1 mM fluorescent dye) was purged with N_2 (23 mLn min^{-1}) or CO_2 (15 mLn min^{-1}) to flush the product gases. The gas flow rate was controlled with a mass flow controller (calibrated for normal

conditions: 0°C, 1 atm). In the case of the N_2 purge, the catholyte had a low CO_2 saturation because the N_2 stripped dissolved CO_2 from the electrolyte. In the case of the CO_2 purge, the catholyte had a high CO_2 saturation because CO_2 bubbles were brought into close contact with the liquid.

Process parameter 2: Reynolds number / Catholyte flow rate

We set the hydraulic Reynolds number in the catholyte channel, Re, to 5 or 50 by adjusting the liquid flow rate of the peristaltic pump, F_{L}, to 0.9 mL min^{-1} or 9.0 mL min^{-1}. We note that this Reynolds number differs from the gas bubble Reynolds number Re_{B} from the main paper and the Reynolds number along a plate electrode, Re_H, used in **Section 3.3**. The hydraulic Reynolds number in the catholyte channel, Re, is calculated with (S1). The hydraulic diameter of the channel is d_{H} in mm. The superficial liquid velocity is u in m s^{-1}. We approximate the kinematic viscosity of the catholyte, ν, with the corresponding value of water at 20°C (ν = 10^{-6} m^2 s^{-1}).[4]

$$Re = \frac{d_{\mathrm{H}} \cdot u}{\nu} \quad \text{(S1)}$$

The value of d_{H} is a function of the channel width, W, in mm and depth, D, in mm (S2).

$$d_{\mathrm{H}} = 2 \cdot \frac{W \cdot D}{W + D} = 2 \cdot \frac{8\ \mathrm{mm}}{2\ \mathrm{mm} + 4\ \mathrm{mm}} = 2.67\ \mathrm{mm} \quad \text{(S2)}$$

$$u = \frac{F_{\mathrm{L}}}{W \cdot D} \quad \text{(S3)}$$

Process parameter 3: Current density

After setting the purge gas flow rate and the liquid flow rate, we used a potentiostat (XP20, Ivium technologies, Netherlands) to set three galvanostatic current density steps (−10, −50, and −100 mA cm^{-2}).

Steady state measurements

The system was given 20 min to equilibrate after setting each process parameter set. Then we recorded the cell potential with the potentiostat. For the experiments with CO_2-saturated electrolyte, the cathode potential was recorded with a Ag/AgCl micro-reference electrode at the catholyte inlet (**Figure S4**). We did not correct the potential for the ohmic resistance of the electrolyte. The bulk pH of the catholyte and anolyte were determined with a pH meter (913 pH Meter, Metrohm AG, Switzerland). The gas flow rate was measured three times with a bubble flow meter to calculate an average gas flow rate for the product mixture, $\dot{V}_{\mathrm{mix}}$. We used a syringe pump to take three gas samples from the product gas stream. These were analyzed with a gas chromatography (GC) system (CompactGC 4.0, Interscience BV, Nederland) to determine the volumetric concentration, C_i, of the following gas species (CO_2, CO, H_2, N_2, O_2, CH_4 , and C_2H_4). No CH_4 or C_2H_4 was detected during our experiments.

The Faradaic efficiency of gas species FE_i (H_2, CO) was calculated from the current density, j, in mA cm^{-2}, the electrode area, A = 1 cm^2, Faraday's constant, F = 96485 As mol^{-1}, the stoichiometric number of electrons exchanged, z_i ($z_i = 2$ for H_2 and CO), and the molar flux of the species, $\dot{N}_i$, in mol s^{-1} (S4).

$$FE_i = \frac{z_i \cdot F \cdot \dot{N}_i}{j \cdot A} \quad \text{(S4)}$$

The molar flux of the species, $\dot{N}_i$, was estimated with the ideal gas law (S5), in which the gas pressure is p = 1 bar, the gas temperature is T = 293.15 K, and the ideal gas constant is R = 8.3145 J K^{-1} mol^{-1}.

$$\dot{N}_i = \frac{p \cdot \dot{V}_{\text{mix}} \cdot C_i}{R \cdot T} \quad \text{(S5)}$$

2.2 Fluorescence lifetime imaging microscopy (FLIM)

The FLIM system measured the phase-shift fluorescence lifetime, τ_ϕ, in the sample region of the flow cell (**Figure S5**) using the frequency-domain technique.[5, 6] The diode laser (405 nm) serves as a light source with a modulation frequency of 20 MHz. The laser light passes through a filter and enters the spinning disk confocal unit (Crest V2, CrestOptics, Italy). The microlense disk focuses the incident light onto the Nipkow pinhole disk (70 µm diameter pinholes), which restricts the light path to the imaged section in the focal plane while scanning the sample region.[7] The focused laser light passes through the objective (5x magnification) of the microscope (Zeiss Axiovert 200M, Carl Zeiss AG, Germany) to excite fluorescent dye molecules in the focal plane of the channel. The focal plane was in a depth of 2 mm and had a focal depth of about 520 µm. These probe molecules emit fluorescent light in a lower wavelength spectrum and, depending on their local pH, the phase of the signal is shifted relative to the phase of the excitation light source. The emitted fluorescent light passes back through the spinning disk unit and is reflected by a dichroic mirror. After passing through the emission filter, the emitted light reaches the FLIM camera (Toggel, Lambert Instruments, Netherlands). The FLIM camera has a resolution of 512 x 470 pixels, which yields a spatial resolution of 4.76 µm per pixel. We recorded all images with a camera gain of 2 and an exposure time of 75 ms. The camera records 6 images in a row with different phase-shifts. Following the frequency-domain technique, a sine curve is fitted through the 6 different intensities recorded for each pixel, from which the fluorescence lifetime is determined. [5, 6] This yields a total imaging time of 450 ms per FLIM image.

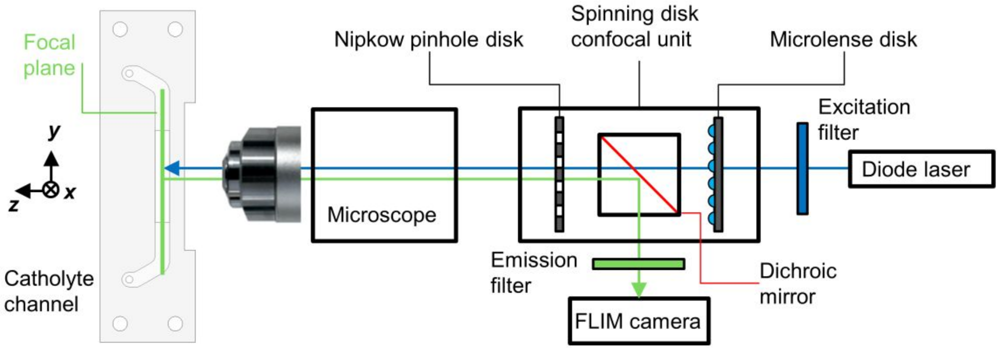

Figure S5: Schematic of FLIM system: Microscope: 5x objective, Modulated diode laser: 405 nm, 20 MHz, 300 mW. FLIM camera: 75 ms exposure time, 6 phase shift images, 2x camera gain.

We calibrated the FLIM system with an in-line titration setup (**Figure S6**). The dye and K_2SO_4 concentration were the same as for the CO_2 electrolysis experiments. We used a low phosphate buffer concentration of 0.0001 M to minimize the influence of phosphate ions on τ_ϕ.[8] The electrolyte was continuously purged with N_2 to prevent ambient CO_2 from dissolving in the electrolyte and influencing the pH. We recirculated the electrolyte through the flow cell (**Figure S3**) to record fluorescence lifetime images for a range of pH values. The pH of the electrolyte was adjusted by incrementally adding base solution to the electrolyte with a syringe pump. The base solution had the same dye and K_2SO_4 concentration to keep the corresponding concentrations in the bulk of the electrolyte constant. After a stabilization period of 2 min, we measured the pH with a pH meter and recorded the FLIM image. The average τ_ϕ in the center of the channel was calculated with the image processing software LIFA (Lambert Instruments, Netherlands) (**Figure S6**).

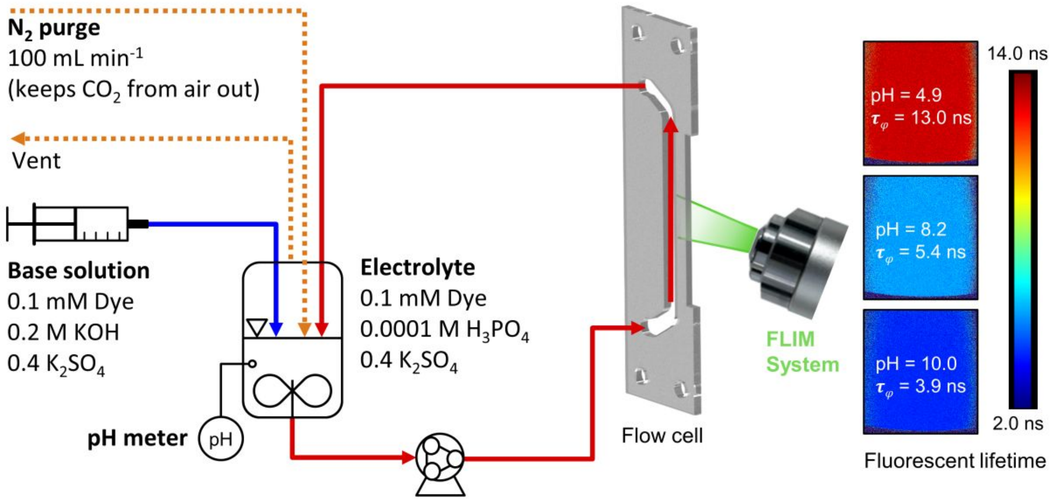

Figure S6: FLIM calibration produce: In-line titration coupled with the FLIM system.

We used a quinolinium dye[8, 9] to obtain the calibration curve for the pH and τ_ϕ (**Figure S7**). The curve has a τ_ϕ-plateau in the acidic region, which makes it impossible to distinguish the pH between 3.0 and 5.5. The dye is most sensitive to changes in τ_ϕ for the pH region between 6.3 and 9. We kept the optical FLIM parameters of the calibration and the CO_2 electrolysis experiments constant to minimize systematic errors.

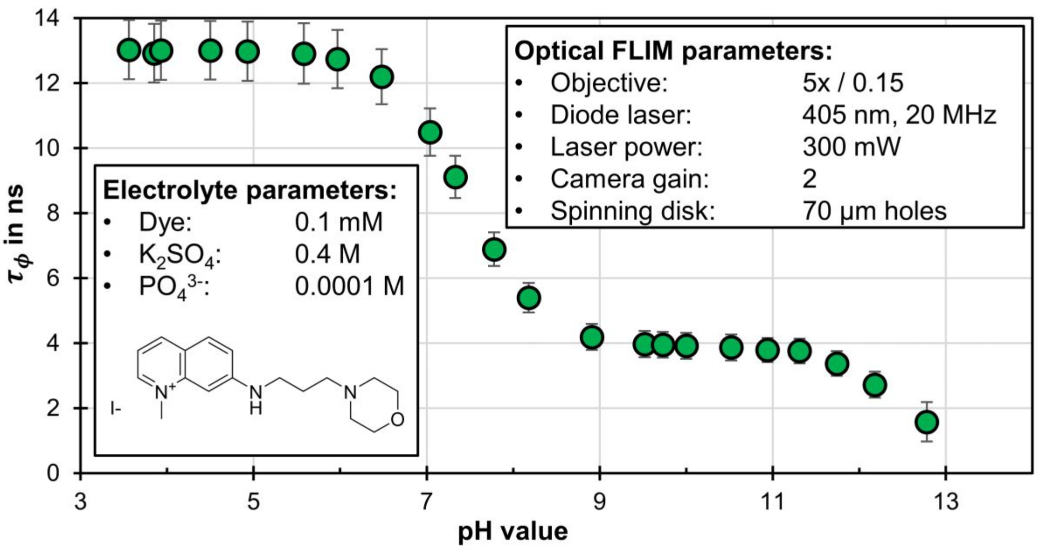

Figure S7: Calibration curve to calculate pH from the phase-shift fluorescent lifetime, τ_ϕ.

The electrochemical stability of the fluorescent dye is discussed in **Figure S8**. Using a similar dye from the 1-methyl-7-amino-quinolinium fluorophore family (Compound 2b in Bleeker *et al.*).[8], we carried out a series of cyclic voltammetry scans (**Figure S8 a**). The dye undergoes a reversible redox reaction 0.5 V and 1 V vs. SHE. The highly reproducible peak reduction current suggests that dye is not decomposing irreversibly into degradation products of the molecule. We assume the dye used in this study (Compound 2c in Bleeker *et al.*).[8] exhibits a similar electrochemical stability. In addition, we sampled the bulk fluorescence intensity from a random selection of FLIM images over the course of a CO_2 electrolysis run of this study (**Figure S8 b**). The dye does not show a decay in intensity over the course of electrolysis while a cathode potential of −0.9 V vs. SHE or less was applied. The variation in intensity is due to changes in the catholyte pH due to process conditions.

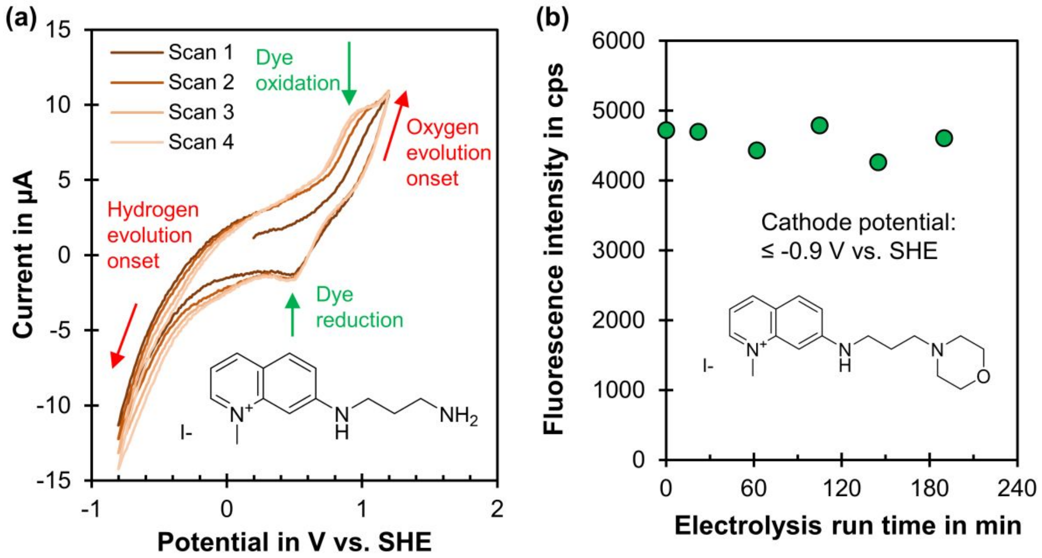

Figure S8: (a) Cyclic voltammetry scans for a member of 1-methyl-7-amino-quinolinium fluorophore family (Compound 2b in Bleeker *et al.*).[8] (b) Bulk fluorescence intensity in counts per second (cps) measured over the course of the CO_2-saturated electrolysis run of this study (Compound 2c in Bleeker *et al.*).[8]

The geometry of the catholyte flow channel and the alignment with the microscope of the FLIM system are shown in **Figure S9**. The straight channel segment with a length of 7.5 mm was left without active area (**Figure S9 a**) to ensure a fully developed hydrodynamic flow profile. The microscope was focused on the outer channel wall (z_0-plane) as a reference point (**Figure S9 b**). Then the control software of the microscope was used to move the focal plane to the center of the channel depth in z-direction (z_2-plane) to carry out the imaging.

To process the FLIM images, we determined the wall coordinates of the channel with intensity images (**Figure S9 c**). The sides of the fluorescence lifetime images were cropped with these coordinates. The PET gaskets forming the wall of the channel show a lower fluorescence lifetime than the bulk of the electrolyte, which most likely originates from the fluorescence of the gasket material.[10] We assume that the gaskets exhibit a constant fluorescence lifetime, τ_{ϕ}, of about 11.5 ns. According to the calibration curve (**Figure S7**), this phenomenon leads to a systematic error, which lets the edges of the image appear to be close to pH 6. Therefore, the local pH near the walls in our images is overestimated at low pH (< 6) and underestimated at high pH (> 6).

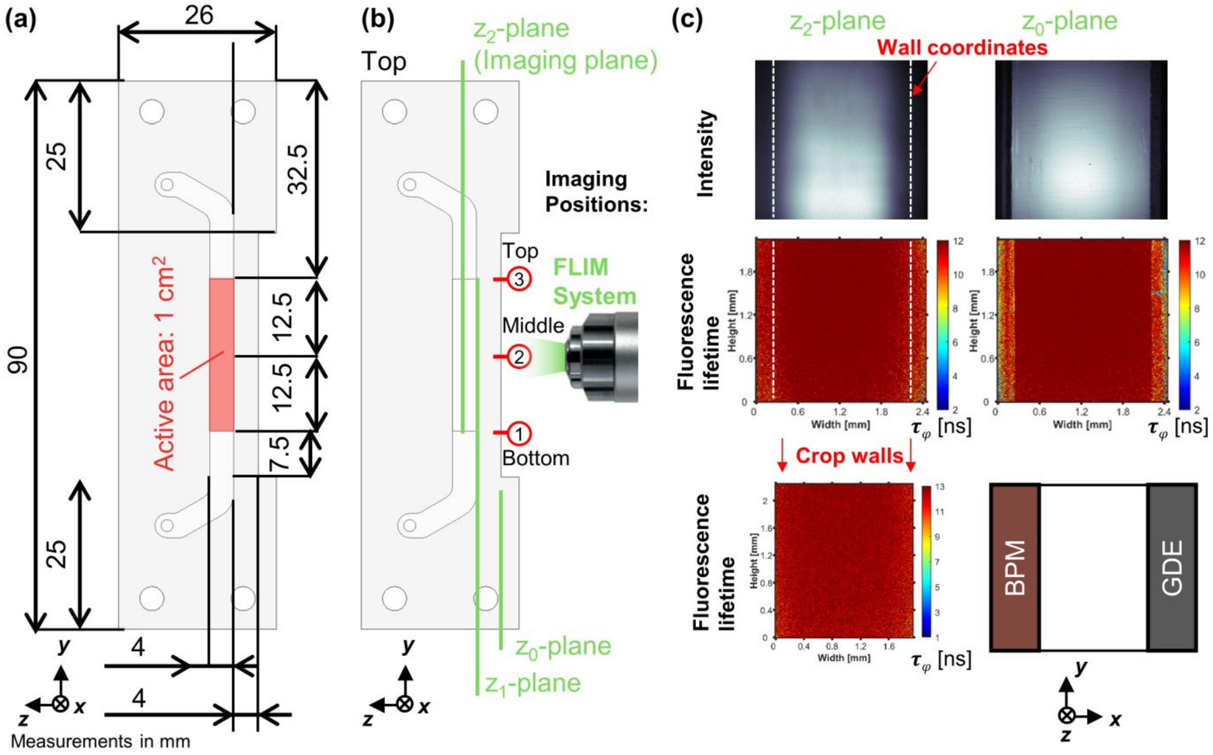

Figure S9: Flow channel geometry and FLIM procedure. (a) Geometry of flow cell. (b) Location of the three imaging positions (1): Bottom, (2): Middle, (3): Top. The outer channel wall (z_0) was used as a focusing reference. The imaging plane (z_2) was located in the middle of the channel depth (2 mm). (c) FLIM data processing: Intensity and phase-shift fluorescence lifetime, τ_ϕ, images of the 0.4 M K_2SO_4 catholyte with 0.1 mM fluorescent quinolonium dye.

During CO_2 electrolysis, we recorded 5 – 10 images at each channel height (**Figure S9 b**) for each process parameter set. Of these images, we selected examples with small amounts of bubble coverage and a clear view of the catholyte channel. These were then further processed with Matlab (Version R2019a). After cropping the walls, the fluorescent lifetime images were converted to 2D pH profiles (**Figure S10**) with the calibration curve. Typically, the local pH value close to the GDE (right side of image) rises because the electrochemical reactions release OH^- ions. We calculated 1D pH profiles by averaging the pH value over the y-axis of the 2D segment. For these 1D profiles, $pH_{avg}(x)$ is the *y*-averaged value over the channel width. The corresponding standard error is $\sigma_{pH}(x)$. The minimum pH of the profile is indicated by pH_{min}; the maximum is indicated by pH_{max}.

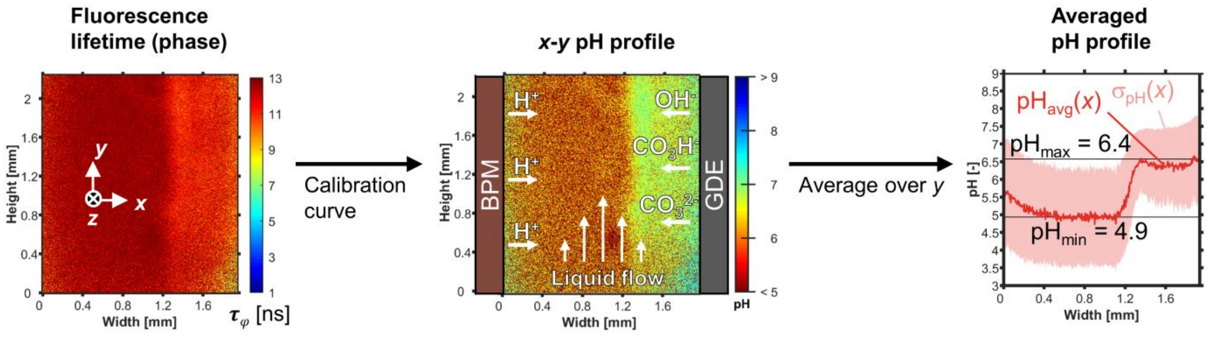

Figure S10: FLIM image processing. The pH profile, $pH_{avg}(x)$, was averaged over the height of the channel segment shown in the upper panel. The shaded red area indicates the standard deviation of the pH value, $\sigma_{pH}(x)$. The minimum value of $pH_{avg}(x)$ is pH_{min}. The maximum value of $pH_{avg}(x)$ is pH_{max}.

The intensity and corresponding fluorescent lifetime images show examples for different effects of gas bubble evolution (**Figure S11**). Typically, gas bubbles form at the surface of the GDE or the BPM (**Figure S11 a**). They

grow until they reach a certain diameter and are released into the flow. This leaves the electrode surface uncovered for a while until the growth cycle starts again. Gas bubbles reduce the intensity of the fluorescence signal even if they are outside of the focal plane. The fluorescent lifetime, τ_{ϕ}, of the focal plane can still be measured as long as there is a sufficient signal-to-noise ratio.

This means that τ_{ϕ} is still available in proximity to growing, stagnant bubbles only casting partial shadows (**Figure S11 a**). However, the intensity close to the GDE often decreases because of intense shadows. In addition to bubbles forming at the surface, these shadows are caused by a lower fluorescence emission of the dye at high pH.[8] As a result, intense shadows cause a noisy signal close to the GDE (**Figure S11 a**). Another phenomenon of the gas bubble evolution are small, moving bubbles (**Figure S11 b**), which follow the flow of the catholyte. These bubbles typically just reduce the intensity slightly, so that τ_{ϕ} experiences only little noise. Occasionally smaller bubbles coalesce and form large, moving bubbles (**Figure S11 c**) taking up most of the channel cross-section. These bubbles cast intense shadows leading to a poor signal-to-noise ratio.

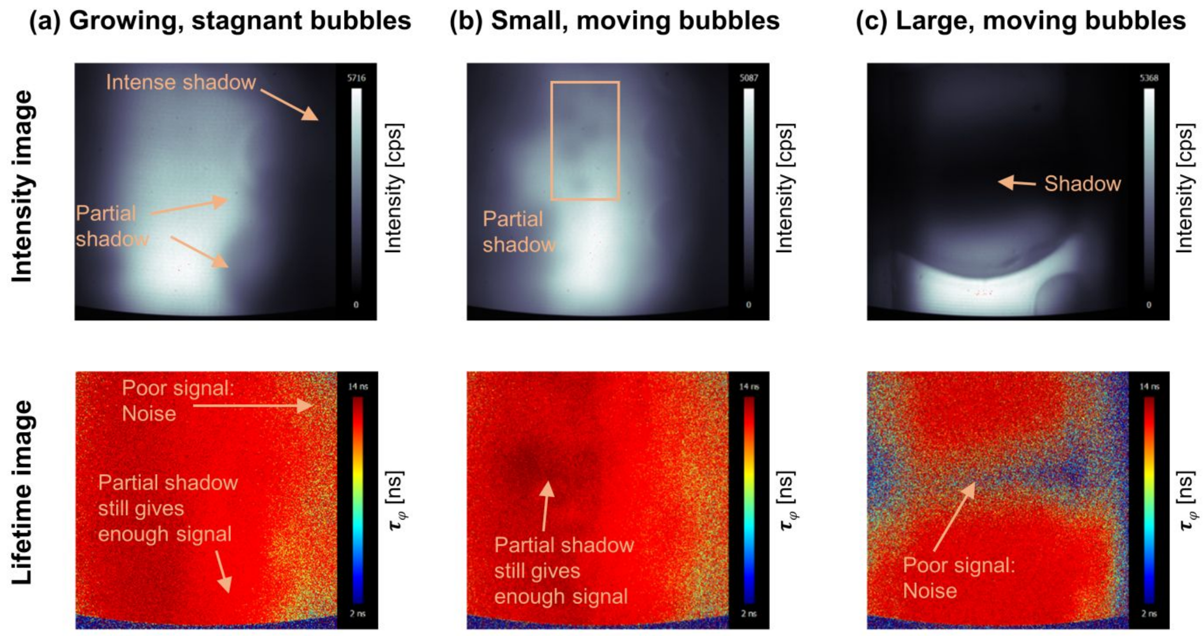

Figure S11: FLIM imaging of catholyte channel with bubble formation. Top: Intensity images. Bottom: corresponding fluorescence lifetime images. The channel walls were not cropped for these example images. (a) Growing bubbles at the GDE surface (b) Small, moving bubbles in the bulk of the catholyte flow (c) Large, moving bubble filling out the cross-section of the channel.

3. Supplementary results and discussion

This section contains an explanation of the calculations used for the results and discussion section of the main article. It further includes the pH profiles for all parameter sets.

3.1 Comparison of bubble evolution for N_2-purged catholyte at Re = 5

Gas evolution at the surface of an electrode can influence the potential in multiple ways. First, gas bubbles forming at the surface of an electrode can increase the activation overpotential by partially blocking the electrocatalytic surface area. If a constant current is supplied to the electrode, the local current density through the remaining active area rises, which results in a higher overpotential. Second, bubbles introduce additional ohmic resistance in the electrolyte by reducing the cross-section available for ion conduction. Third, bubbles can reduce the concentration overpotential when being released from a surface because their movement induces convective mass transfer from the bulk of the electrolyte.[11]

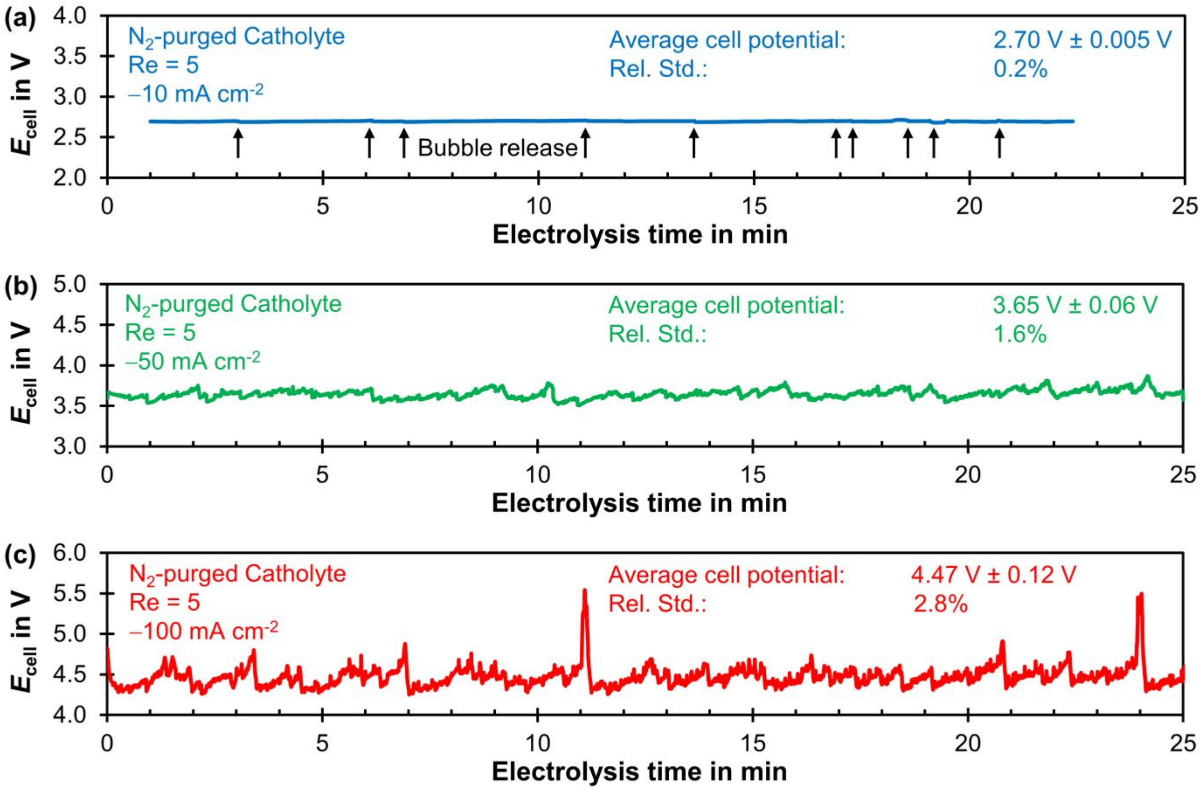

Figure S12: Comparison of bubble evolution for Re = 5 and N_2-purged catholyte: Cell potential, E_{cell}, as a function of electrolysis time at the specified current density, j. **(a)** $j = -10$ mA cm^{-2}. The arrows indicate drops in potential due to bubble release. **(b)** $j = -50$ mA cm^{-2} **(c)** $j = -100$ mA cm^{-2}. The average E_{cell} ± its sample standard deviation was evaluated from 1 min after electrolysis start to the end of the current density step. The relative standard deviation (Rel. Std.) is the ratio of the sample standard deviation of E_{cell} to the average of E_{cell}.

Bubble evolution leads periodic increases and drops in potential at micro[12] and macro electrodes.[13] We use the fluctuations in E_{cell} to compare the gas evolution as a function of j (**Figure S12**). At $j = -10$ mA cm^{-2}, very little gas evolution takes place. The drops in E_{cell} (indicated by arrows) show that during an electrolysis time of 22 min only 10 bubbles were released from the anode and cathode (**Figure S12 a**). Further, E_{cell} varies less than 0.2% around its average value of 2.7 V (**Figure S12 a**). We therefore conclude that bubble evolution and plays an insignificant role at this current density. In contrast, the amplitude and frequency of potential fluctuations are much stronger at higher j (**Figure S12 b and c**).

3.2 Calculation of unbuffered pH limit

We calculate the unbuffered pH limit, $pH_{\mathrm{unbuffered}}$, by making the following assumptions:

- OH^- is perfectly mixed across the channel width (x-direction)
- No neutralization with H^+ occurs
- No homogenous buffering reactions with CO_2 take place

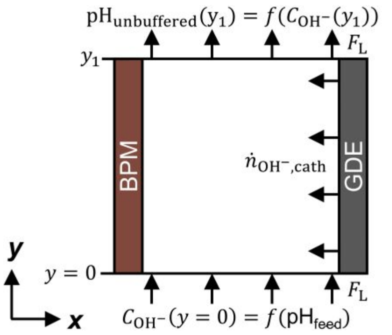

Figure S13: Mass balance to calculate the unbuffered pH limit, $\mathrm{pH_{unbuffered}}$, over a channel segment with the height y_1. The molar flux of OH^- produced in the reaction is $\dot{n}_{\mathrm{OH^-,cath}}$.

To set up the mass balance, we further assume that the catholyte flows through the channel segment at a volumetric flow rate, F_L, (**Figure S13**). At the inlet ($y = 0$), the initial concentration of $C_{\mathrm{OH^-}}$ is determined by $\mathrm{pH_{feed}}$ using (S6).

$$C_{\mathrm{OH^-}} = 10^{-\mathrm{pOH}} = 10^{-(14-\mathrm{pH})} \tag{S6}$$

We consider the pH increase over the height of a channel segment with the height y_1 (**Figure S13**). For example, to calculate $\mathrm{pH_{unbuffered}}$ at the middle of the channel height, we set y_1 to 1.25 cm. The electrochemical reaction releases a uniform flux of OH^-, $\dot{n}_{\mathrm{OH^-,cath}}$, in mol cm^{-2} s^{-1}, which depends on j according to (S7).

$$\dot{n}_{\mathrm{OH^-,cath}} = \frac{j}{F} \tag{S7}$$

Over the considered electrode height of y_1 and depth of $D = 0.4\ \mathrm{cm}$ (z-direction), the cumulative flux $\dot{N}_{\mathrm{OH^-,cath}}(y_1)$ is released according to (S8).

$$\dot{N}_{\mathrm{OH^-,cath}}(y_1) = \dot{n}_{\mathrm{OH^-,cath}} \cdot D \cdot y_1 \tag{S8}$$

The released OH^- mixes with the catholyte stream and increases the concentration of OH^- according to (S9).

$$C_{\mathrm{OH^-}}(y_1) = C_{\mathrm{OH^-}}(y_0) + \frac{\dot{N}_{\mathrm{OH^-,cath}}(y_1)}{F_\mathrm{L}} \tag{S9}$$

The value of $\mathrm{pH_{unbuffered}}$ is then calculated with (S10).

$$\mathrm{pH_{unbuffered}}(y_1) = 14 - (-\log_{10} C_{\mathrm{OH^-}}(y_1)) \tag{S10}$$

3.3 CO_2 bubble evolution at bipolar membrane

The electrolysis process splits water inside the bipolar membrane (BPM) and transports H^+ ions to the interface with the catholyte. If the catholyte feed is already saturated with CO_2, the reduction of local pH caused by the H^+ ions can lead to an oversaturation with dissolved CO_2 due to the carbonate equilibrium ($H^+ + HCO_3^- \leftrightarrow H_2CO_3 \leftrightarrow CO_{2(aq.)} + H_2O$). An example of CO_2 bubbles forming at the surface of the BPM is given in **Figure S14**.

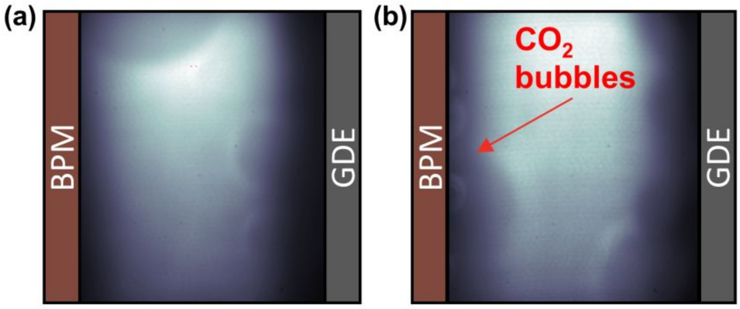

Figure S14: Intensity images showing bubble evolution at middle of channel height (y = 12.5 mm). (a) N_2-purged catholyte, $Re =$ 50, j = –50 mA cm^{-2}. (b) CO_2-saturated catholyte, $Re =$ 50, j = –50 mA cm^{-2}: CO_2 bubble formation at surface of bipolar membrane (BPM).

3.4 Catholyte Reynolds number interferes with bubble-induced mixing

We calculate the limiting partial current density for CO $j_{\mathrm{CO,lim}}$ with (S11). This quantity corresponds to the convective mass transfer of CO_2 from the bulk of the flowing catholyte to the electrode surface, $\dot{N}_{\mathrm{CO_2,conv}}$. The number of electrons transferred in the reaction is $z_{\mathrm{CO}} = 2$.

$$j_{\mathrm{CO,lim}} = \frac{z_{\mathrm{CO}} \cdot F \cdot \dot{N}_{\mathrm{CO_2,conv}}}{A} \quad \text{(S11)}$$

We calculate $\dot{N}_{\mathrm{CO_2,conv}}$ in mol s^{-1} with (S12), in which k_{conv} is the convective mass transfer coefficient in cm s^{-1}. The mass transfer is driven by the CO_2 concentration gradient, $\Delta C_{\mathrm{CO_2}}$, which takes a maximum value of $C_{\mathrm{CO_2,bulk}}$ if the CO_2 concentration at the electrode surface drops to zero. The bulk concentration of CO_2 at 25°C is approximated with $C_{\mathrm{CO_2,bulk}} = 34\ \mathrm{mM}$.[14]

$$\dot{N}_{\mathrm{CO_2,conv}} = A \cdot k_{\mathrm{conv}} \cdot \Delta C_{\mathrm{CO_2}} \approx A \cdot k_{\mathrm{conv}} \cdot C_{\mathrm{CO_2,bulk}} \quad \text{(S12)}$$

The value of k_{conv} is determined by the correlation (S13) of the Sherwood number, Sh, with the Reynolds number over the height of the electrode, Re_H, and the Schmidt number, Sc.[15] The height of the electrode, H, is 2.5 cm. The diffusion coefficient for CO_2 in water at 25°C is $D_{\mathrm{CO_2}} = 1.9 \cdot 10^{-5}\ \mathrm{cm^2 s^{-1}}$.[14]

$$k_{\mathrm{conv}} = \frac{D_{\mathrm{CO_2}} \cdot Sh}{H} = 0.664 \cdot \frac{D_{\mathrm{CO_2}}}{H} \cdot Re_{\mathrm{H}}^{0.5} \cdot Sc^{0.333} \quad \text{(S13)}$$

We calculate the Reynolds number over the height of the electrode, Re_H, with (S14). We note that this Reynolds number is different than the hydraulic Reynolds number, Re, defined by (S1). The superficial liquid velocity, u, is given by (S3). We approximate the kinematic viscosity of the catholyte, ν, with the corresponding value of water at 20°C (ν = 10^{-6} m^2 s^{-1}).[4] The value of Sc is given by (S15).

$$Re_H = \frac{H \cdot u}{\nu} \quad \text{(S14)}$$

$$Sc = \frac{\nu}{D_{CO_2}} = 532 \tag{S15}$$

The resulting values for k_{conv} and $j_{CO,lim}$ are listed for hydraulic Reynolds numbers of $Re = 5$ and $Re = 50$ in **Table S1**.

Table S1: CO_2 mass transfer through forced convection. The hydraulic Reynolds number is Re. The volumetric catholyte flow rate is F_L. The superficial liquid velocity is u. The Reynolds number along the height of the electrode is Re_H. The convective mass transfer coefficient is k_{conv}. The corresponding limiting current density for CO is $j_{CO,lim}$.

Re	F_L	u	Re_H	k_{conv}	$j_{CO,lim}$
-	mL min^{-1}	cm s^{-1}	-	cm s^{-1}	mA cm^{-2}
5	0.9	0.19	46	$2.8 \cdot 10^{-4}$	1.8
50	9.0	1.88	464	$8.8 \cdot 10^{-4}$	5.8

The effect of increasing the Reynolds number on the FE_{CO} is shown in **Figure S15**.

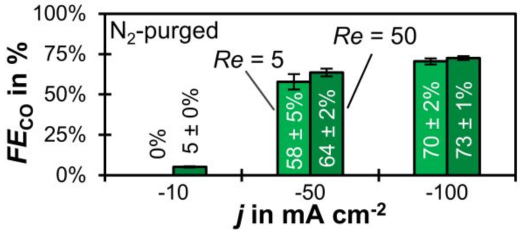

Figure S15: Effect of Reynolds number in catholyte channel, Re. FE_{CO} as a function of j and Re for N_2-purged catholyte. The error bars represent the estimated standard errors of three GC injections.

3.5 Calculation of CO_2 buffering capacity

We estimate the nominal pH buffer capacity of the dissolved CO_2, $\dot{N}_{CO_2,buff}$, in mol s^{-1} with (S16). This value corresponds to the molar flux of OH^- that the dissolved CO_2 could absorb through the formation of CO_3^{2-}. For simplification, we neglect equilibrium reactions and assume that every dissolved molecule of CO_2 absorbs 2 molecules of OH^-.

$$\dot{N}_{CO_2,buff} = F_L \cdot 2 \cdot c_{CO_2,bulk} \tag{S16}$$

We compare this buffering capacity with the total molar flux of OH^- formed in electrochemical reactions (S17). We neglect the consumption of CO_2 through the CO_2R reaction. This gives us a best case scenario for the ratio of $\dot{N}_{CO_2,buff}$ to $\dot{N}_{OH^-,cath}$ (**Table S2**).

$$\dot{N}_{OH^-,cath} = \frac{j \cdot A}{F} \tag{S17}$$

Table S2: Nominal buffering capacity of dissolved CO_2. The hydraulic Reynolds number is Re. The volumetric catholyte flow rate is F_L. The current density is j. The nominal pH buffer capacity of the dissolved CO_2 is $\dot{N}_{CO_2,buff}$. The total molar flux of OH^- formed in electrochemical reactions is $\dot{N}_{OH^-,cath}$.

Re	F_L	j	$N_{CO_2,buff}$	$N_{OH^-,cath}$	$\frac{N_{CO_2,buff}}{N_{OH^-,cath}}$
-	mL min^{-1}	mA cm^{-2}	mol s^{-1}	mol s^{-1}	-
5	0.9	50	$9.9 \cdot 10^{-7}$	$5.2 \cdot 10^{-7}$	191%
5	0.9	100	$9.9 \cdot 10^{-7}$	$1.0 \cdot 10^{-6}$	96%
50	9.0	50	$9.9 \cdot 10^{-6}$	$5.2 \cdot 10^{-7}$	1910%

50	9.0	100	$9.9 \cdot 10^{-6}$	$1.0 \cdot 10^{-6}$	955%

3.6 Complete set of pH profiles

The following section shows the 2D and 1D pH profiles for each parameter set (**Figure S16** – **Figure S23**).

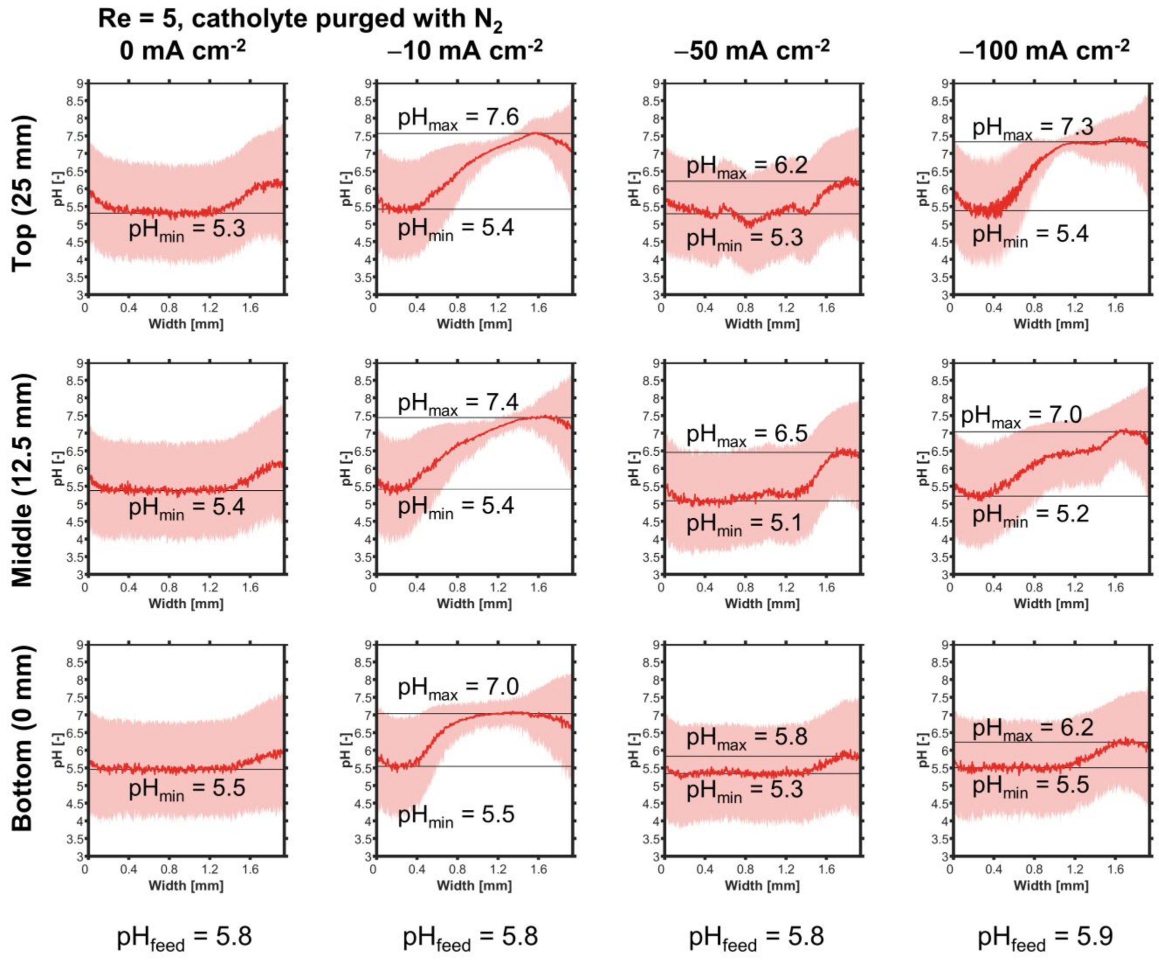

Figure S16: 1D pH profiles for Re = 5 and N_2-purged catholyte. The pH profile, pH_{avg}, was averaged over the height of the channel segment shown in the upper panel. The shaded red area indicates the standard deviation of the pH value. The minimum value of pH_{avg} is pH_{min}. The maximum value of pH_{avg} is pH_{max}. The pH value of the catholyte feed, pH_{feed}, was measured with a pH meter.

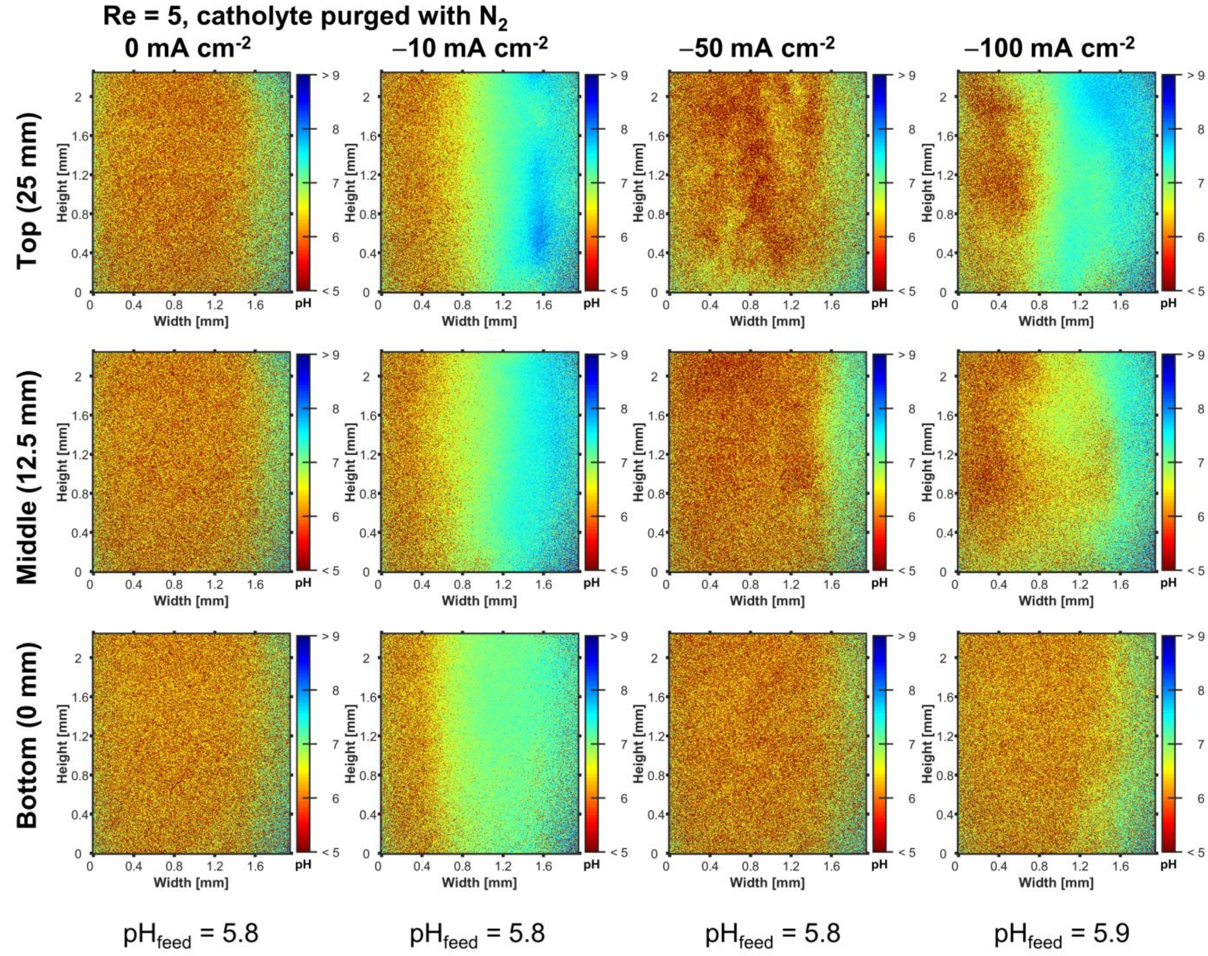

Figure S17: 2D pH profiles for Re = 5 and N_2-purged catholyte. The pH value of the catholyte feed, pH_{feed}, was measured with a pH meter.

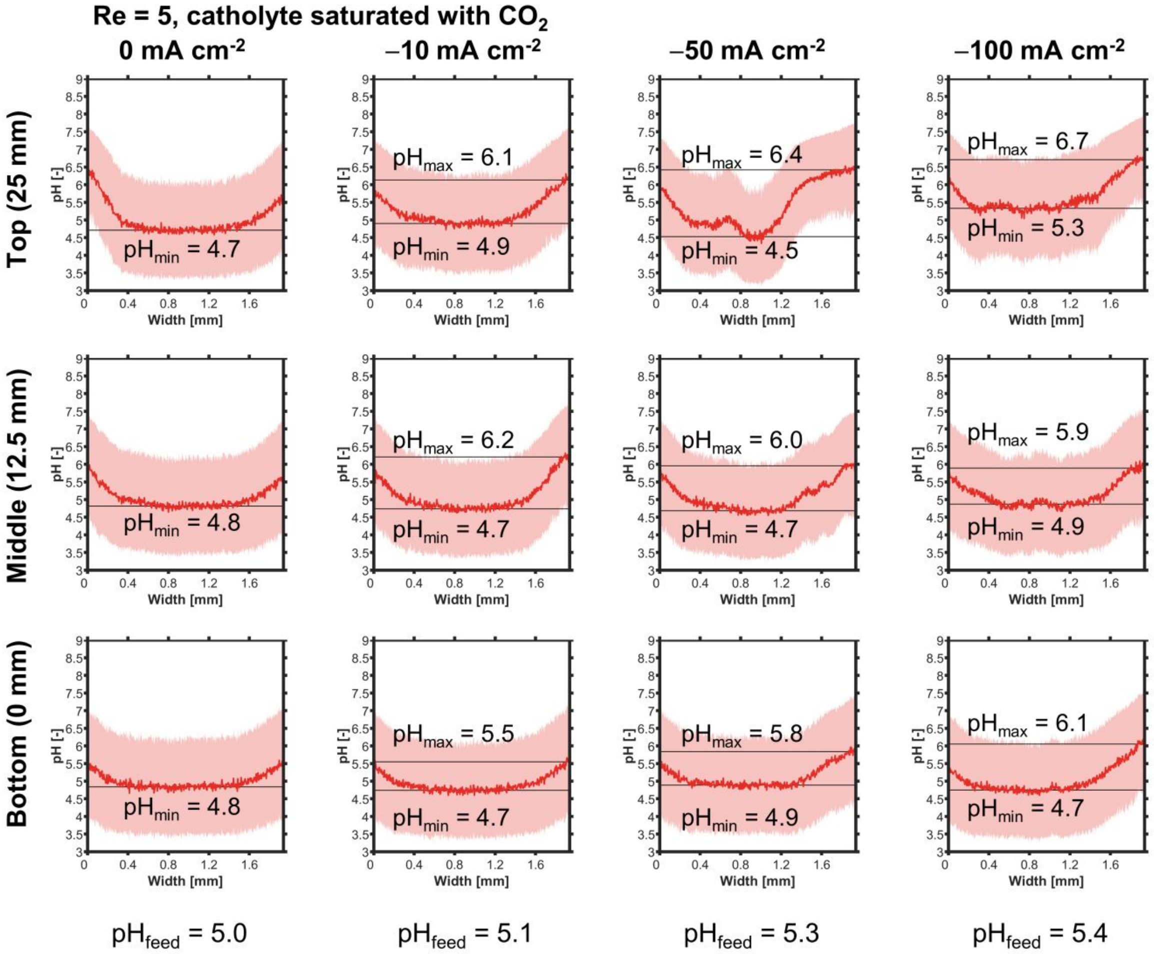

Figure S18: 1D pH profiles for Re = 5 and CO_2-saturated catholyte. The pH profile, pH_{avg}, was averaged over the height of the channel segment shown in the upper panel. The shaded red area indicates the standard deviation of the pH value. The minimum value of pH_{avg} is pH_{min}. The maximum value of pH_{avg} is pH_{max}. The pH value of the catholyte feed, pH_{feed}, was measured with a pH meter.

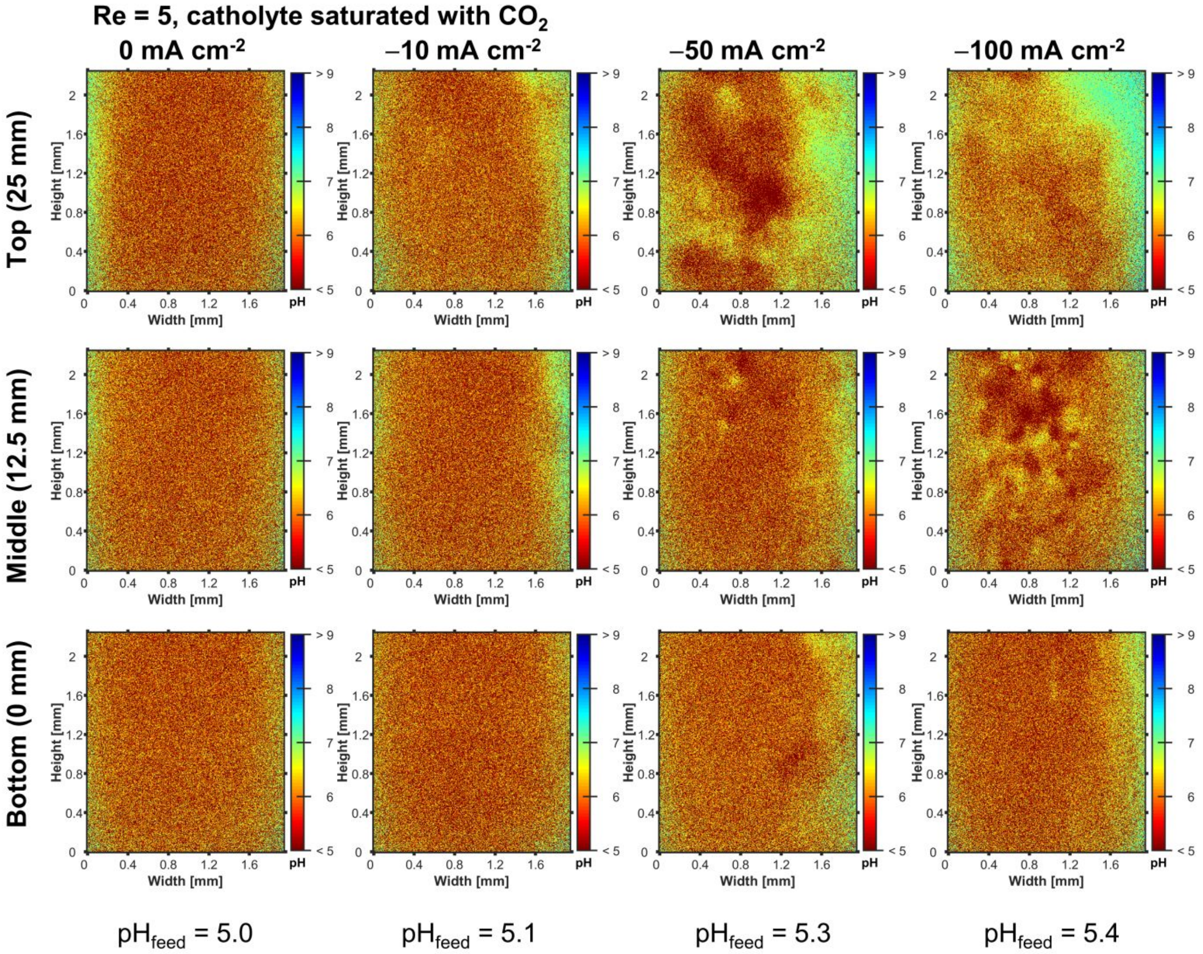

Figure S19: 2D pH profiles for Re = 5 and CO_2-saturated catholyte. The pH value of the catholyte feed, pH_{feed}, was measured with a pH meter.

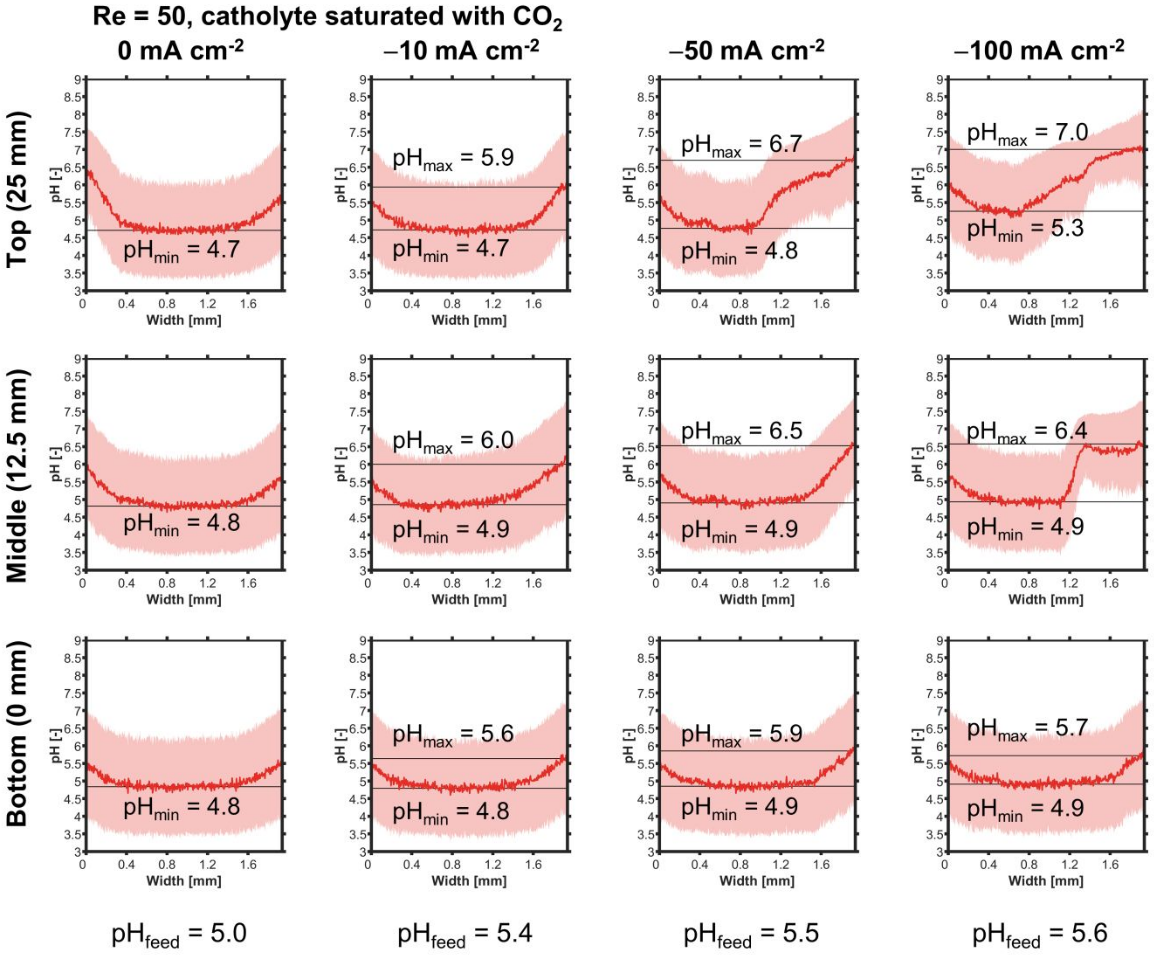

Figure S20: 1D pH profiles for Re = 50 and CO_2-saturated catholyte. The pH profile, pH_{avg}, was averaged over the height of the channel segment shown in the upper panel. The shaded red area indicates the standard deviation of the pH value. The minimum value of pH_{avg} is pH_{min}. The maximum value of pH_{avg} is pH_{max}. The pH value of the catholyte feed, pH_{feed}, was measured with a pH meter.

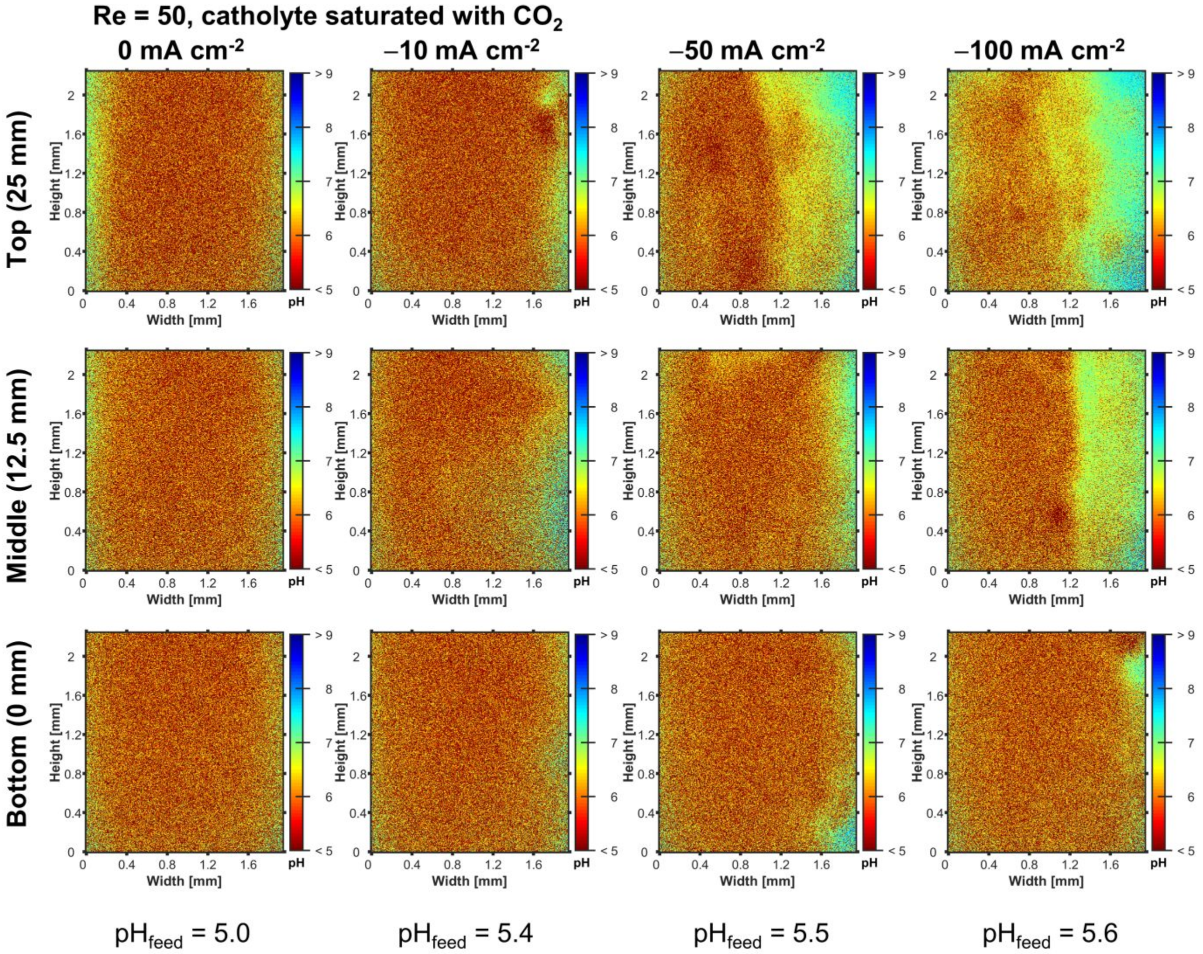

Figure S21: 2D pH profiles for Re = 50 and CO_2-saturated catholyte. The pH value of the catholyte feed, pH_{feed}, was measured with a pH meter.

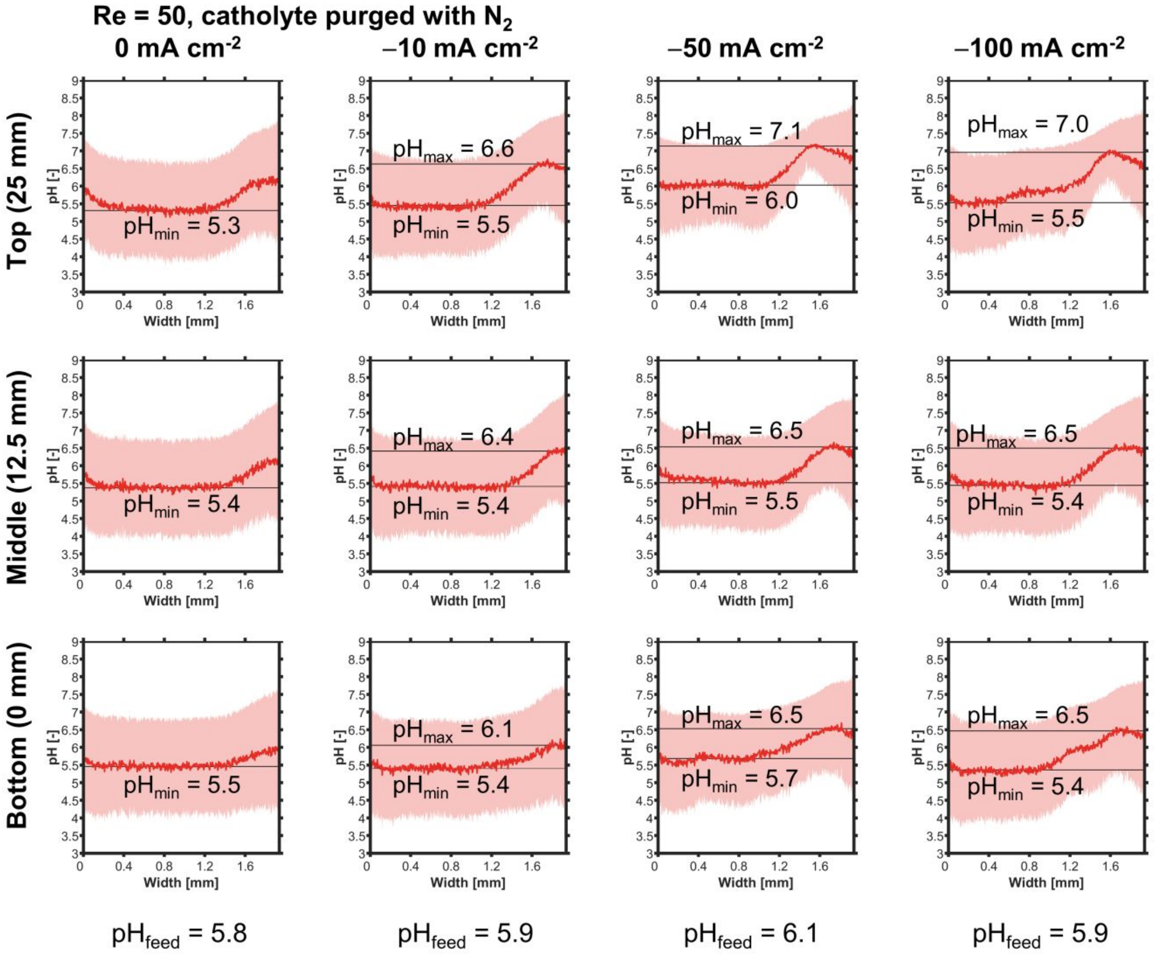

Figure S22: 1D pH profiles for Re = 50 and N_2-purged catholyte. The pH profile, pH_{avg}, was averaged over the height of the channel segment shown in the upper panel. The shaded red area indicates the standard deviation of the pH value. The minimum value of pH_{avg} is pH_{min}. The maximum value of pH_{avg} is pH_{max}. The pH value of the catholyte feed, pH_{feed}, was measured with a pH meter.

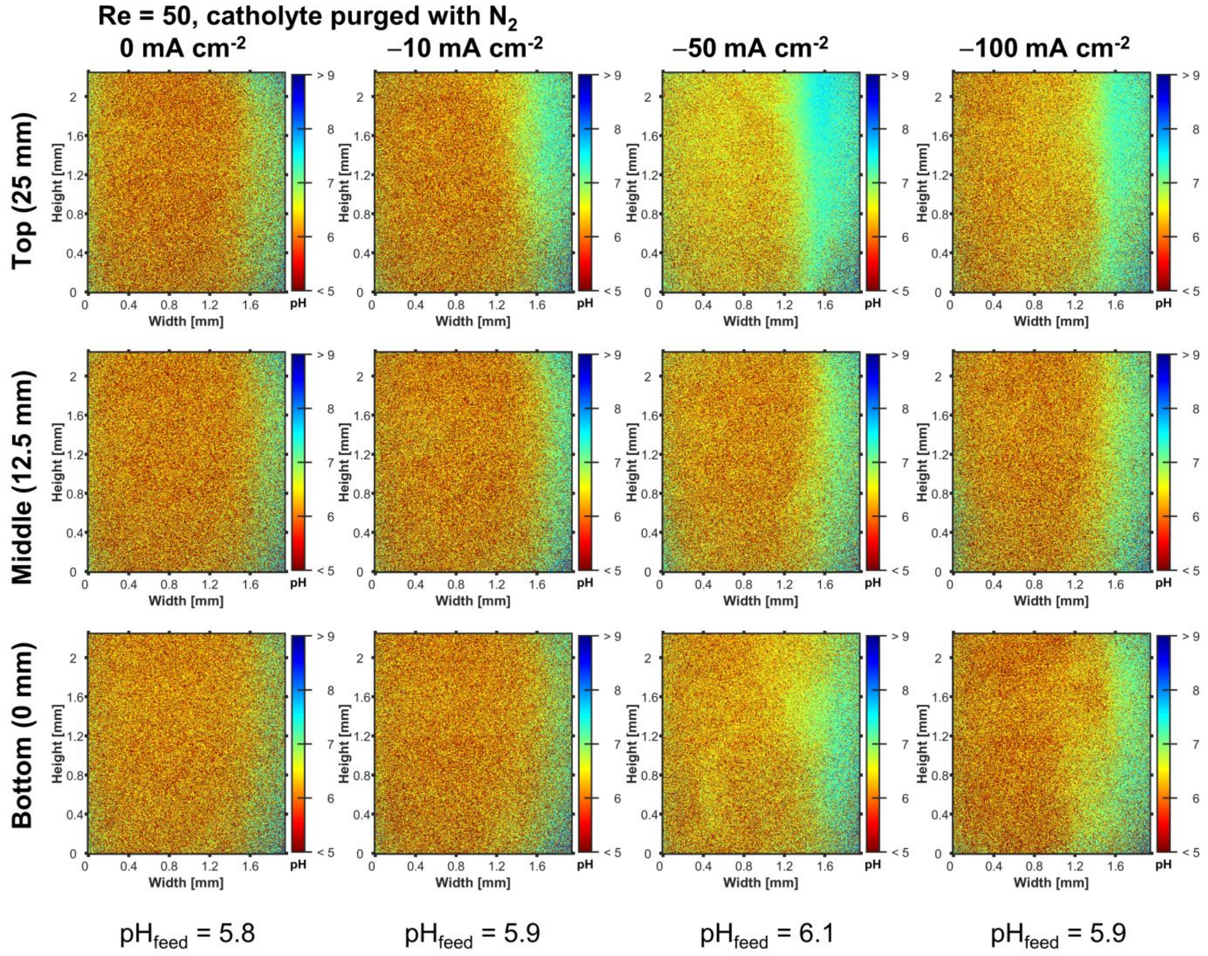

Figure S23: 2D pH profiles for Re = 50 and CO_2-saturated catholyte. The pH value of the catholyte feed, pH_{feed}, was measured with a pH meter.

References

1. Baumgartner, L.M., C.I. Koopman, A. Forner-Cuenca, and D.A. Vermaas, *Narrow Pressure Stability Window of Gas Diffusion Electrodes Limits the Scale-up of Co2 Electrolyzers.* ACS Sustainable Chemistry & Engineering, 2022. **10**(14): p. 4683-4693.
2. Duarte, M., B. De Mot, J. Hereijgers, and T. Breugelmans, *Electrochemical Reduction of Co2: Effect of Convective Co2 Supply in Gas Diffusion Electrodes.* ChemElectroChem, 2019. **6**(22): p. 5596-5602.
3. Baumgartner, L.M., C.I. Koopman, A. Forner-Cuenca, and D.A. Vermaas, *When Flooding Is Not Catastrophic – Woven Gas Diffusion Electrodes Enable Stable Co2 Electrolysis.* ACS Applied Energy Materials, 2022(5): p. 15125-15135.
4. Lienhard, J.H., *A Heat Transfer Textbook*. 2019: Dover Publications.
5. van Munster, E.B. and T.W.J. Gadella, *Fluorescence Lifetime Imaging Microscopy (Flim)*, in *Microscopy Techniques*, J. Rietdorf, Editor. 2005, Springer Berlin Heidelberg: Berlin, Heidelberg. p. 143-175.
6. Lakowicz, J.R., *Principles of Fluorescence Spectroscopy*. 2013: Springer Science & Business Media.
7. Tanaami, T., S. Otsuki, N. Tomosada, Y. Kosugi, M. Shimizu, and H. Ishida, *High-Speed 1-Frame/Ms Scanning Confocal Microscope with a Microlens and Nipkow Disks.* Applied optics, 2002. **41**(22): p. 4704-4708.
8. Bleeker, J., A.P. Kahn, L.M. Baumgartner, F.C. Grozema, D.A. Vermaas, and W.F. Jager, *Quinolinium-Based Fluorescent Probes for Dynamic Ph Monitoring in Aqueous Media at High Ph Using Fluorescence Lifetime Imaging (Flim).* ACS sensors, 2023.
9. Jager, W.F., T.S. Hammink, O. van den Berg, and F.C. Grozema, *Highly Sensitive Water-Soluble Fluorescent Ph Sensors Based on the 7-Amino-1-Methylquinolinium Chromophore.* The Journal of Organic Chemistry, 2010. **75**(7): p. 2169-2178.
10. Ouchi, I., R. Miyamura, M. Sakaguchi, S. Hosaka, and M. Kitagawa, *Excitation and Emission Spectra of Polyethylene Terephthalate and Polyethylene 2, 6-Naphthalate Films.* Polymers for Advanced Technologies, 1999. **10**(3): p. 195-198.
11. Angulo, A., P. van der Linde, H. Gardeniers, M. Modestino, and D.F. Rivas, *Influence of Bubbles on the Energy Conversion Efficiency of Electrochemical Reactors.* Joule, 2020. **4**(3): p. 555-579.

12. Peñas, P., P. van der Linde, W. Vijselaar, D. van der Meer, D. Lohse, J. Huskens, H. Gardeniers, M.A. Modestino, and D.F. Rivas, *Decoupling Gas Evolution from Water-Splitting Electrodes.* Journal of The Electrochemical Society, 2019. **166**(15): p. H769.
13. Angulo, A.E., D. Frey, and M.A. Modestino, *Understanding Bubble-Induced Overpotential Losses in Multiphase Flow Electrochemical Reactors.* Energy & Fuels, 2022. **36**(14): p. 7908-7914.
14. Gupta, N., M. Gattrell, and B. MacDougall, *Calculation for the Cathode Surface Concentrations in the Electrochemical Reduction of Co 2 in Khco 3 Solutions.* Journal of applied electrochemistry, 2006. **36**(2): p. 161-172.
15. Burdyny, T., P.J. Graham, Y. Pang, C.-T. Dinh, M. Liu, E.H. Sargent, and D. Sinton, *Nanomorphology-Enhanced Gas-Evolution Intensifies Co2 Reduction Electrochemistry.* ACS Sustainable Chemistry & Engineering, 2017. **5**(5): p. 4031-4040.
